# Major Traumatic Injury and Exposure to Mitochondrial-Derived Damage-Associated Molecular Patterns Promotes Neutrophil Survival Accompanied by Stabilisation of the Anti-Apoptotic Protein Mcl-1

**DOI:** 10.3390/cells14100754

**Published:** 2025-05-21

**Authors:** Thomas Nicholson, Michael Macleod, Antonio Belli, Janet M. Lord, Jon Hazeldine

**Affiliations:** 1 Department of Inflammation and Ageing, School of Infection, Inflammation and Immunology, College of Medicine and Health, University of Birmingham, Birmingham B15 2TT, UK; t.a.nicholson@bham.ac.uk (T.N.); mxm1283@bham.ac.uk (M.M.); a.belli@bham.ac.uk (A.B.); j.m.lord@bham.ac.uk (J.M.L.); 2 National Institute for Health Research Surgical Reconstruction and Microbiology Research Centre, Queen Elizabeth Hospital Birmingham, Birmingham B15 2TT, UK; 3 MRC-Versus Arthritis Centre for Musculoskeletal Ageing Research, University of Birmingham, Birmingham B15 2TT, UK

**Keywords:** apoptosis, critical care, injury, mitochondrial-derived damage-associated molecular patterns, neutrophils, trauma

## Abstract

Traumatic injury leads to an extension of the half-life of circulating neutrophils. However, how quickly neutrophil apoptosis is delayed post-injury is currently unknown, as are the underlying mechanisms and factors that promote this extension of lifespan. During the ultra-early (≤1 h) and acute (4–12 and 48–72 h) post-injury phases, we collected blood samples from 73 adult trauma patients. Following ex vivo culture, neutrophil apoptosis was measured, alongside caspase-3 activation and expression of the anti-apoptotic protein Mcl-1. To identify factors that may promote neutrophil survival post-trauma, neutrophils from healthy controls (HCs) were cultured with mitochondrial-derived damage-associated molecular patterns (mtDAMPs) or mitochondrial DNA (mtDNA). Accompanied by reduced mitochondrial membrane depolarisation, delayed Mcl-1 turnover, and reduced caspase-3 activation, the ex vivo lifespan of neutrophils from trauma patients was significantly enhanced in a protein synthesis-independent manner within minutes to hours after injury. Neutrophils from HCs exhibited delayed apoptosis when cultured in media supplemented with trauma patient serum, which occurred alongside stabilisation of Mcl-1. Culturing HCs neutrophils with mtDAMPs or mtDNA significantly delayed apoptosis rates, promoted stabilisation of Mcl-1, and reduced caspase-3 activation. The release of mtDAMPs from damaged tissue may drive post-trauma immune dysregulation by promoting the survival of dysfunctional neutrophils.

## 1. Introduction

Neutrophil dysfunction is a well-described consequence of major traumatic and thermal injury [1,2,3,4,5,6]. Characterised by increased expression of adhesion molecules, enhanced basal migration, and the production of reactive oxygen species, an immediate state of neutrophil hyperactivity is accompanied by impaired functional responses to ex vivo stimulation [2,4,6,7,8,9,10,11,12,13,14]. Associated with these functional changes is a trauma-induced delay in neutrophil apoptosis. By promoting cellular and tissue injury at remote organs, the extended lifespan of dysregulated neutrophils has been proposed to contribute to the development of such secondary complications as multiple organ dysfunction syndrome (MODS), multiple organ failure (MOF), and sepsis [15,16,17,18].

Performed across multiple timepoints, spanning from hospital admission to day 11 post-injury, results from ex vivo culture assays have shown that neutrophils isolated from polytrauma, blunt/penetrative trauma, traumatically brain injured, and burn patients are resistant to the induction of spontaneous apoptosis [8,15,17,18,19,20,21,22]. Negatively associated with physiological markers of injury severity and organ function, such as the Acute Physiology and Chronic Health Evaluation (APACHE) score and the multiple organ dysfunction score [17], this trauma-induced extension of neutrophil lifespan has been attributed to impaired activation of the intrinsic and extrinsic apoptosis pathways [16,18,21,23]. Mediated by the mitochondria, the induction of apoptosis is controlled by the opposing actions of members of the Bcl-2 family of pro- and anti-apoptotic proteins that together regulate mitochondrial membrane permeability. By promoting mitochondrial permeabilization, pro-apoptotic proteins, such as Bcl-2-associated X protein (Bax) and Bid, trigger activation of caspases 3 and 9 [24,25]. Counteracting the actions of pro-apoptotic Bcl-2 proteins are the anti-apoptotic proteins myeloid cell leukaemia 1 (Mcl-1), Bcl-xL, and A1. Preventing mitochondrial outer membrane permeabilization and the release of cytochrome C, Mcl-1 binds to, and deactivates, the pro-apoptotic complex formed by the Bcl-2 proteins Bak and Bax [26,27]. A substrate of the proteasome and caspase-3, Mcl-1 is a short-lived protein, with an estimated half-life of 1–5 h [26]. However, when neutrophils are exposed to inflammatory factors and microbial-derived products such as chemokines, growth factors, and lipopolysaccharide, the turnover rate of Mcl-1 is delayed, which results in an extension of lifespan [28,29].

Studies that have investigated the molecular processes that underpin the trauma- induced extension of neutrophil half-life have focused predominantly upon changes in the expression of the Bcl-2 family proteins. Alongside a significant reduction in Bax, thermal and traumatic injuries have been reported to lead to an upregulation of Mcl-1 and Bcl-xL expression, which coincides with reduced activation of caspases 3 and 9 [16,21,30]. Notably, these changes in pro- and anti-apoptotic proteins were detected in neutrophils that were analysed immediately post-isolation [21,30]. Thus, without ex vivo culture, the mechanisms by which trauma modulates the expression of Bcl-2 family proteins is unclear. For example, is the post-injury upregulation of Mcl-1 a consequence of increased transcription and/or translation, or is it a result of delayed turnover?

Demonstrating the presence of pro-survival factors in the circulation of injured patients, several studies have shown that exposing neutrophils isolated from healthy volunteers to plasma or serum obtained from traumatic and thermally-injured subjects significantly extends their lifespan ex vivo [15,16,17,21,22,31,32]. Identifying a role for granulocyte macrophage colony stimulating factor (GM-CSF) in promoting this survival effect, the pre-treatment of patient sera with neutralising antibodies against this growth factor resulted in higher rates of spontaneous neutrophil apoptosis and increased Mcl-1 turnover [21,22]. However, the pro-survival effect of patient sera was not totally eradicated, meaning that other factors that extend neutrophil lifespan are present in the circulation post-injury.

Released from injured tissue and activated immune cells in the minutes, hours, and days following major trauma, mitochondrial-derived damage-associated molecular patterns (mtDAMPs) are a heterogeneous collection of proteins, lipids, and DNA [33,34,35,36]. Suggesting a potential link between mtDAMPs and delayed neutrophil apoptosis post-trauma, Bhagirath et al. demonstrated that neutrophils isolated from healthy donors exhibited significantly increased viability following ex vivo culture with mitochondrial-derived DNA (mtDNA) [37]. However, the mechanisms underlying this extension of lifespan were not investigated.

Here, via the analysis of blood samples acquired from trauma patients across the ultra-early (≤1 h) and acute (4–72 h) post-injury phases, we have investigated the molecular mechanisms that underly the post-trauma extension of neutrophil lifespan. Complementing these studies, we have built upon previous research [37] to examine, at the mechanistic level, how in vitro exposure to mtDAMPs delays the induction of apoptosis in neutrophils isolated from healthy volunteers.

## 2. Materials and Methods

### 2.1. Study Design and Setting

This manuscript presents data acquired from subjects enrolled in the Brain Biomarkers after Trauma Study (BBATS), a prospective observational cohort study of adult trauma patients conducted at a single major trauma centre site in the UK (University Hospitals Birmingham NHS Foundation Trust, Birmingham). Ethical approval was granted by the North Wales Research Ethics Committee—West (REC reference: 13/WA/0399, Protocol Number: RG_13-164).

Patient enrolment began in the pre-hospital setting, where, on a 24/7 basis between November 2017 and May 2023, emergency care teams acquired blood samples from adult trauma patients (≥18 years) with a suspected injury severity score (ISS) ≥8 within 1 h of injury (defined as the time of phone call to emergency services). Information regarding study exclusion criteria and the process of obtaining informed patient consent has been described previously [12].

### 2.2. Clinical Data Collection

Patient and injury details were obtained prospectively from electronic and physical medical records. Data collected included patient age, sex, mechanism of injury, time of injury, severity of injury (Injury Severity Score (ISS) and Glasgow Coma Scale (GCS)). Patient mortality, intensive care unit (ICU) and hospital-free days (calculated as 30 minus the number of days the patient stayed in ICU and hospital, respectively) were extracted from electronic clinical records. Patients who died in the hospital or ICU setting within 30 days of admission were assigned a score of 0.

### 2.3. Blood Sampling

In the pre-hospital setting, venous blood samples were acquired during the intravenous cannulation of patients or by venepuncture. Samples were stored at room temperature (RT) during transportation to hospital, where upon arrival, they were collected for analysis within 1 h of deposition by a single laboratory researcher. Additional blood samples were acquired from patients 4–12 and 48–72 h post-injury. At each study timepoint, a total blood volume of 24 mL was collected into BD Vacutainers^®^ (BD Biosciences, Oxford, UK) containing ethylenediaminetetraacetic acid, 1/10 volume of 3.2% trisodium citrate, or z-serum clotting activator. Data obtained from trauma patients who received steroid treatment were not included in the final analysis.

A total of 67 adults (mean age 37 years, range 20–83 years) served as a cohort of healthy controls (HCs). HCs were recruited in accordance with the Declaration of Helsinki, with ethical approval was granted by the University of Birmingham Research Ethics Committee (Ref: ERN_12-1184). HCs enrolled into this study were not taking any regular medication for a diagnosed illness and had not experienced an episode of infection in the two weeks prior to sampling. HCs were excluded from the study if they were taking any medication that would modify immune responses, such as steroids.

### 2.4. Preparation of MtDAMPs, MtDNA, and Serum

MtDNA and mtDAMPs were prepared from mitochondria isolated from the K562 tumour cell line (ATCC^®^, Teddington, Middlesex, UK), as described previously [38]. The purity and concentration of mtDNA preparations, as well as the protein content of mtDAMPs, were determined by spectrophotometry (Nanodrop 2000; Thermo Fisher Scientific, Paisley, UK).

Serum was prepared from blood collected into BD vacutainers containing z-serum clotting activator. Following a 30 min incubation at RT, blood samples were centrifuged at 1620× *g* for 10 min at 4 °C, after which serum was aliquoted and stored at −80 °C.

### 2.5. Neutrophil Isolation and Treatment

Neutrophils were isolated from the blood samples of trauma patients and HCs via Percoll density gradient centrifugation (Scientific Lab Supplies, Nottingham, UK). Following re-suspension in RPMI-1640 media supplemented with 2 mM L-glutamine, 100 U/mL penicillin, 100 µg/mL streptomycin (GPS; Sigma-Aldrich, Dorset, UK), and 10% heat-inactivated foetal calf serum (hereafter referred to as complete medium (CM)), the purity of the neutrophil preparations was determined using a Sysmex XN-1000 haematology analyser (Sysmex UK, Milton Keynes, UK). Routinely, neutrophils comprised ≥99% of the isolated cell population.

For ex vivo experiments that compared the rates of spontaneous and accelerated apoptosis between neutrophils isolated from trauma patients and HCs, neutrophils were cultured (37 °C/5% CO_2_) for 6 or 18 h in the presence or absence of the protein synthesis inhibitor cycloheximide (CHX, 50 µM, Sigma-Aldrich) or the transcription inhibitor actinomycin D (Act D, 1 µM, Sigma-Aldrich). To examine the effect of the “trauma microenvironment” on neutrophil spontaneous apoptosis, neutrophils isolated from HCs were cultured for 18 h in RPMI-1640 media supplemented with GPS and either 10% autologous serum or serum obtained from trauma patients 4–12 h post-injury. To study the effect of mtDAMP and mtDNA treatment on neutrophil lifespan, neutrophils isolated from HCs were cultured for 30–360 min (37 °C/5% CO_2_) with 40 or 100 µg/mL mtDAMPs or mtDNA in the presence or absence of 50 µM CHX or 1 µM Act D. To rule out any potential effects of contaminating endotoxin within our mtDAMPs or mtDNA preparations, neutrophils were pre-treated for 1 h with 10 or 20 µg/mL polymyxin B (Sigma-Aldrich).

To inhibit the proteasome, neutrophils isolated from HCs were treated for 1 h with 1 µM epoxomicin (Sigma-Aldrich) or 5 µM lactacystin (Sigma-Aldrich). To inhibit caspase-3, neutrophils isolated from HCs were treated for 1 h with 50 or 100 µM Z-DEVD-FMK (Bio-Techne, Abingdon, Oxford, UK). To block signalling through formyl peptide receptor-1 (FPR-1), neutrophils isolated from HCs were treated for 1 h (37 °C/5% CO_2_) with 1 µM cyclosporin H (CsH; Abcam, Cambridge, UK) prior to mtDAMP stimulation.

### 2.6. Annexin V and Propidium Iodide (PI) Staining

The neutrophils (2 × 10^5^ in CM) were pelleted by centrifugation (250× *g*, 5 min, 4 °C) and re-suspended in 198 µL of ice cold 1x Annexin V binding buffer (BD Pharmingen, Oxford, UK). The neutrophils were then stained in the dark for 20 min on ice with 2 µL Annexin V-FITC (BD Pharmingen), after which the samples were centrifuged (250× *g*, 5 min, 4 °C) and re-suspended in 200 µL of ice cold 1X Annexin V binding buffer. Following transfer to polypropylene FACS tubes, the neutrophils were stained with 2 µg/mL PI, and the samples analysed immediately on an Accuri C6 flow cytometer (BD Biosciences, Oxford, UK) or CyAn^ADP^ bench top cytometer (Dako, Cambridgeshire, UK). Based on forward scatter/sideward scatter properties, 10,000 neutrophils were gated, and the percentage presented using an Annexin V^−^ PI^−^ phenotype (defined as “live” cells) was recorded.

### 2.7. Measurement of Mitochondrial Membrane Potential

Mitochondrial membrane potential in neutrophils isolated from trauma patients and HCs was examined using 5,5′,6,6′-tetrachloro-1,1′,3,3′-tetraethylbenzimidazolylcarbocyanine iodide (JC-1; Fisher Scientific, Loughborough, UK), a membrane-permeant dye that accumulates in energised mitochondria. Neutrophils (1 × 10^6^ in CM) were cultured for 6 h (37 °C/5% CO_2_) in the presence or absence of 40 or 100 µg/mL mtDAMPs, 40 µg/mL mtDNA, or 50 µM CHX. Post-treatment, cells were washed in phosphate buffered saline (PBS; 250× *g*, 5 min, RT), and the pellet was re-suspended in 500 nM JC-1. Following a 30 min incubation (37 °C/5% CO_2_), cells were washed and re-suspended in PBS prior to analysis on an Accuri C6 flow cytometer (BD Biosciences) or a CyAn^ADP^ bench top cytometer (Dako). A total of 10,000 neutrophils were analysed, and the mean fluorescence intensity (MFI) values of the FL1 and FL2 channels were recorded. Data are presented as the ratio of FL1:FL2 MFI values. A representative flow cytometry plot depicting the measurement of mitochondrial membrane potential is shown in Supplementary Figure S1.

### 2.8. Assessment of Caspase-3 Activation by Flow Cytometry

Following a 6 h treatment with 100 µg/mL mtDAMPs or vehicle control in the presence or absence of 50 µM CHX, neutrophils isolated from HCs (4 × 10^5^ in CM) were pelleted by centrifugation (250× *g*, 5 min, RT) and fixed in 50 µL of fixation media (medium A; Fisher Scientific) for 20 min at RT. After a single wash (250× *g*, 5 min, RT) in PBS, the cells were permeabilized in 50 µL of permeabilization media (medium B; Life Technologies, Chesire, UK) for 30 min at RT. Following one wash in PBS, cells were cultured for 1 h at RT in a 50% goat serum/PBS mix (*v*/*v*), washed once in PBS, and stained for 30 min at RT with 5 µg/mL FITC rabbit anti-active caspase-3 (BD Pharmingen) or its concentration-matched isotype control (Invitrogen, Carlsbad, CA, USA). After one wash in PBS, samples were transferred to polypropylene FACS tubes and analysed on an Accuri C6 flow cytometer. A total of 10,000 neutrophils were gated, and both the percentage of active caspase-3-expressing cells and MFI values were recorded. A representative flow cytometry plot depicting the measurement of caspase-3 activation is shown in Supplementary Figure S2.

### 2.9. Western Blotting

Protein lysates from neutrophils isolated from trauma patients and HCs were prepared in hot sodium dodecyl sulphate (SDS) sample buffer (4% SDS (*v*/*v*), 0.1 M dithiothreitol, 20% glycerol (*v*/*v*), 0.0625 M Tris–HCL, and 0.004% bromophenol blue (*w*/*v*)). Lysates were generated from freshly isolated neutrophils (2–5 × 10^6^) and neutrophils subjected to the following conditions: (i) 18 h ex vivo culture in CM, (ii) 18 h ex vivo culture in the presence of 10% autologous serum or serum isolated from trauma patients 4–12 h post-injury, (iii) 1, 2, or 3 h treatment with 50 µM CHX, (iv) 3 and/or 6 h culture with 40 µg/mL mtDAMPs or 40 µg/mL mtDNA, (v) 2 and 3 h co-culture with 40 µg/mL mtDAMPs and 50 µM CHX, (vi) 1 h treatment with 1 µM epoxomicin prior to a 2 or 3 h culture with 50 µM CHX, or (vii) 1 h pre-treatment with 50 or 100 µM Z-DEVD-FMK, followed by a 3 h culture with 50 µM CHX.

Proteins were separated on 10, 12, or 15% SDS-polyacrylamide gels, transferred to polyvinylidene difluoride membranes (Bio-Rad, Hertfordshire, UK), and the membranes were probed overnight at 4 °C with rabbit anti-human antibodies directed against Mcl-1, A1, Bax, or cleaved caspase-3 (Cell Signaling Technology Europe B.V., Leiden, The Netherlands; all diluted 1:1000 in tris-buffered saline containing 0.001% tween (TBST)). Post-incubation, the membranes were washed in TBST and incubated for 1 h at RT with a goat anti-rabbit secondary antibody conjugated to horse radish peroxidase (HRP; diluted 1:4000 in TBST; GE Healthcare, Buckinghamshire, UK). HRP activity was detected using enhanced chemiluminescence (Bio-Rad). To check protein loading, blots were probed with antibodies against β-actin (1:5000, GeneTex, Irvine, CA, USA) or total P38 (1:1000; Cell Signaling Technology). Total P38 was used as the loading control for experiments that involved the treatment of neutrophils with mtDAMPs, given the association of mitochondria with the actin cytoskeleton. Densitometry analysis was performed using Image J fiji-windows-x64 software (National Institutes of Health, Bethesda, MD, USA).

### 2.10. Real-Time PCR (RT-PCR)

For experiments that compared Mcl-1 mRNA levels in neutrophils isolated from trauma patients and HCs, cells were lysed in 1 mL of TRIzol reagent (Life Technologies, Cheshire, UK), and RNA was extracted, as described in the manufacturer’s protocol. Isolated RNA was re-suspended in 30 µL of RNase-free water (Life Technologies), heated at 55 °C for 10 min, and its concentration quantified using a NanoDrop 2000 (Thermo Fisher Scientific). To examine the effect of mtDAMP treatment on Mcl-1 gene transcription, freshly isolated neutrophils (5 × 10^6^) were stimulated for 0, 30, 60, and 120 min with 40 µg/mL mtDAMPs, after which cells were lysed in RLT buffer (Qiagen, Manchester, UK), and total RNA was extracted using an RNeasy Mini kit, according to the manufacturer’s instructions (Qiagen). Extracted RNA exhibited A260/A280 ratios of 1.8–2.0, which was deemed suitable for analysis.

mRNA expression of Mcl-1 was determined, relative to the housekeeping gene 18S, by RT-PCR, using the iTaq^™^ Universal SYBR^®^ Green One-step kit mastermix (Bio-Rad), 5 ng total RNA, and primers (Mcl-1: Forward 5′GGA CAT CAA AAA CGA AGA CG3′, Reverse 5′GCA GCT TTC TTG GTT TAT GG3′. 18S: Forward 5′GTA ACC CGT TGA ACC CCA TT3′, Reverse 5′CCA TCC AAT CGG TAG CG3′). For each RT-qPCR performed, a non-template control consisting of iTaq^™^ Universal SYBR^®^ Green One-step mastermix and gene-specific primers was included to rule out the contamination of PCR reagents. All reactions comprised a total volume of 5 µL and were performed in triplicate. Data were acquired using a Bio-Rad sfx cycler (Bio-Rad) and analysed via the 2^−ΔΔCt^ method using Bio-Rad CFX manager software (Bio-Rad).

### 2.11. Reactive Oxygen Species (ROS) Production

ROS production by neutrophils isolated from HCs was measured using luminol-amplified chemiluminescence. Following a 6 h treatment with vehicle, 50 µM CHX, or 1 µM Act D in the presence or absence of 100 µg/mL mtDAMPs, the neutrophils (2 × 10^6^ in CM) were washed once in PBS (1500× *g*, 2 min, RT) and re-suspended to 1 × 10^6^/mL in Hank’s balanced salt solution (HBSS), supplemented with calcium and magnesium (hereafter referred to as HBSS^+/+^; Gibco, Life Technologies, Cheshire, UK). A total of 100 µL aliquots of neutrophils (1 × 10^5^/mL in HBSS^+/+^) were dispensed into wells of a 96-well white-bottomed flat plate (BD Biosciences), pre-coated with PBS/2% Bovine serum albumin (BSA), that contained 25 µL of luminol (pH 7.3; final concentration 100 µM; Sigma-Aldrich) and 50 µL HBSS^+/+^. The neutrophils were then stimulated with 25 nM Phorbol 12-myristate 13-acetate (PMA), or vehicle control (DMSO), after which ROS generation was assessed at 1 min intervals for 180 min using a Berthold Centro LB 960 luminometer (Berthold Technologies, Hertfordshire, UK). The experiments were performed in quadruplicate, with ROS production measured as relative light units and calculated as the area under the curve (AUC). A representative image derived from a luminol-amplified chemiluminescence assay is shown in Supplementary Figure S3.

### 2.12. Neutrophil Phagocytosis

The phagocytic activity of neutrophils isolated from HCs was assessed using pHrodo Red *E. coli* BioParticles (Invitrogen). Prior to use in phagocytic assays, the bioparticles were opsonised for 1 h (37 °C/5% CO_2_) at a concentration of 1 mg/mL in HBSS^+/+^ containing 20 mM Hepes (pH 7.4) and 10% pooled human serum. Following a 6 h treatment with vehicle, 50 µM CHX, or 1 µM Act D in the presence or absence of 100 µg/mL mtDAMPs, the neutrophils (2 × 10^6^ in CM) were washed once in PBS (1500× *g*, 2 min, RT) and re-suspended at 1 × 10^6^/mL in CM. 100 µL of cell suspension was dispensed into wells of a PBS/2% BSA pre-coated 96-well “U”-bottomed plate, to which 50 µL of bioparticles (1 mg/mL) were added. Following a 60 min incubation (37 °C/5% CO_2_), the samples were washed once in ice-cold PBS/2% BSA and stained for 20 min on ice with 0.11 µg/mL mouse anti-human Allophycocyanin (APC)-conjugated CD15 antibody (BD Pharmingen). Post-incubation, the samples were washed twice in PBS/2% BSA (250× *g*, 5 min, 4 °C) prior to analysis on an Accuri C6 flow cytometer. A total of 10,000 CD15^+^ cells were gated, and both the percentage of bioparticle positive cells and the accompanying MFI values were recorded. To ascertain background fluorescence readings, a negative control sample comprised of neutrophils stimulated on ice with bioparticles for 60 min was included in all experiments. Readings from these samples were subtracted from the results of all test samples. A representative flow cytometry plot depicting the measurement of neutrophil phagocytosis is shown in Supplementary Figure S4.

### 2.13. CD16 Staining

Following a 6 h treatment with vehicle, 50 µM CHX, or 50 µM CHX and 100 µg/mL mtDAMPs, neutrophils isolated from HCs (2 × 10^5^ in CM) were washed once in PBS (250× *g*, 5 min, 4 °C), and the pellets were resuspended in 100 µL PBS containing 4.5 µg/mL of a mouse anti-human CD16 APC-conjugated monoclonal antibody or concentration matched isotype control (BioLegend UK Ltd., London, UK). Following a 20 min incubation on ice, the samples were washed once in PBS (250× *g*, 5 min, 4 °C), and the cells were resuspended in 200 µL PBS prior to flow cytometric analysis on an Accuri C6 flow cytometer. A total of 10,000 neutrophils, gated on forward scatter/sideward scatter properties, were analysed, with both the percentage of CD16^LOW^ and CD16^HIGH^ neutrophils and accompanying MFI values recorded (Supplementary Figure S5).

### 2.14. Morphological Assessment of Neutrophil Apoptosis

Immediately post-isolation, and following a 6 h treatment with 50 µM CHX in the presence or absence of 100 µg/mL mtDAMPs, the morphology of the neutrophils isolated from HCs was assessed using light microscopy. Briefly, freshly isolated, CHX, or CHX and mtDAMP treated neutrophils (2 × 10^5^ in CM) were loaded into Cytospin chambers and centrifuged at RT for 5 min at 300 revolutions per minute in a Cytospin 4 bench top centrifuge (ThermoFisher Scientific). Post-spin, the samples were air dried for 5 min prior to staining with a Reastain Quick Diff Kit, according to the manufacturer’s guidelines (Reagena, Toivala, Finland). The slides were visualised using a Will Wetzlar light microscope at X32 magnification.

### 2.15. Statistical Analyses

Statistical analyses were performed using GraphPad Prism^®^ 10 software (GraphPad Software., Boston, MA, USA). Data distribution was examined using the Kolmogorov–Smirnov or Shapiro–Wilk normality tests. For normally distributed data, paired and unpaired Student *t*-tests, a repeated measures ANOVA with a Bonferroni’s or Dunnett’s multiple comparison post hoc test, a one-way ANOVA with Dunnett’s multiple comparison post hoc test, or a Pearson’s correlation test were performed. For non-normally distributed data, a Wilcoxon matched-pairs signed rank test, a Friedman test with Dunn’s multiple comparison post hoc test, a Kruskal–Wallis with Dunn’s multiple comparison post hoc test, a Mann–Whitney U test, or Spearman’s correlation test was performed. Statistical significance was set at *p* ≤ 0.05.

## 3. Results

### 3.1. Patient Demographics

A total of 73 adult trauma patients (65 male, 8 female), with a mean age of 41 years (range 19–95 years) and a mean injury severity score of 25 (range 9–66), were enrolled into the study (Table 1). Road traffic collisions were the predominant mechanism of injury, with the mean time of pre-hospital blood sampling of 40 min post-injury (range 14–60 min).

### 3.2. Neutrophil Apoptosis Is Delayed Post-Trauma

**Figure 1:**
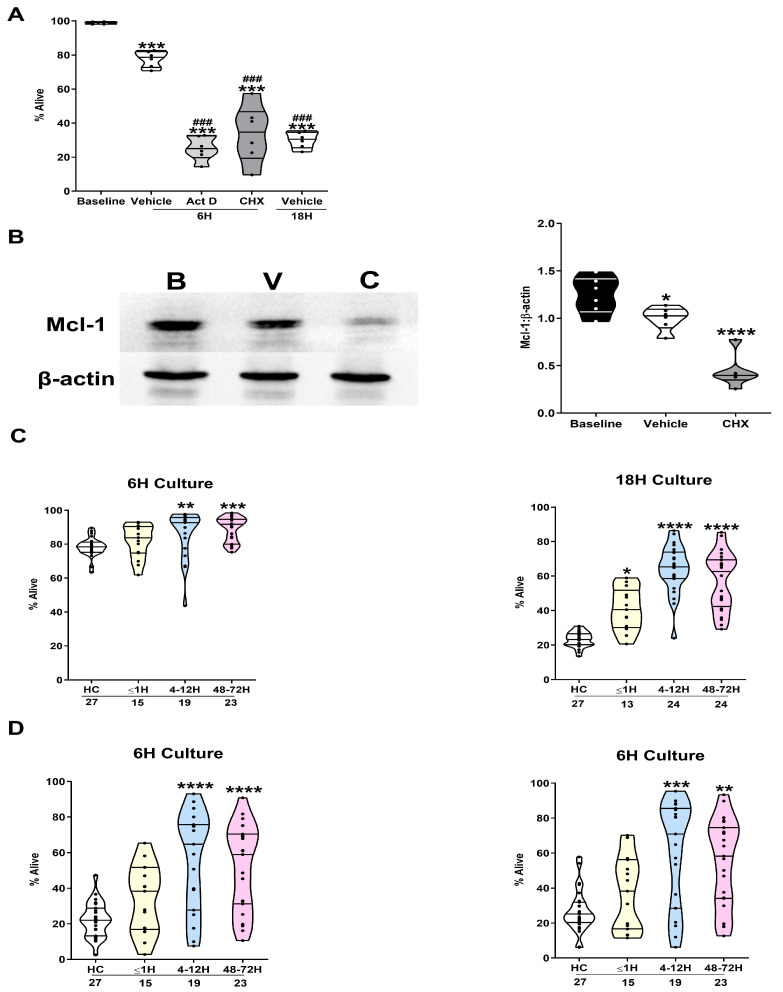
Traumatic injury delays neutrophil apoptosis in vitro. (**A**) Apoptosis, assessed by annexin V/PI staining, was measured for neutrophils isolated from HCs (n = 6) immediately post-isolation (baseline) or following a 6 or 18 h culture in the presence or absence of vehicle control (DMSO), the transcription inhibitor Act D (1 µM), or the protein synthesis inhibitor CHX (50 µM). Alive cells were defined as Annexin V^−^/PI^−^. *** *p* < 0.0005 vs. baseline; ^###^ *p* < 0.0005 vs. 6 h vehicle. (**B**) Mcl-1 protein expression in neutrophils acquired from HCs immediately post-isolation (baseline) and following a 3 h culture with vehicle (DMSO) or 50 µM CHX. Left panel; representative Western blot: B, baseline; V, vehicle; C, CHX; right panel, collated densitometry data from six independent experiments. * *p* < 0.05; **** *p* < 0.0001 vs. baseline. (**C**) Comparison of spontaneous apoptosis rates between neutrophils isolated from HCs and trauma patients at three post-injury timepoints (≤1, 4–12, and 48–72 h) following a 6 h (left panel) or 18 h (right panel) ex vivo culture. Alive cells were defined as Annexin V^−^/PI^−^ neutrophils. The number of samples analysed is indicated below each timepoint. * *p* < 0.05, ** *p* < 0.005, *** *p* < 0.0005, **** *p* < 0.0001 vs. HCs. (**D**) Comparison of CHX (left panel) and Act D (right panel) induced apoptosis rates between neutrophils isolated from HCs and trauma patients at three post-injury timepoints (≤1, 4–12 and 48–72 h) following a 6 h culture. Alive cells were defined as Annexin V^−^/PI^−^ neutrophils. The number of samples analysed is indicated below each timepoint. ** *p* < 0.005, *** *p* < 0.0005, **** *p* < 0.0001 vs. HCs.

Annexin V and PI staining demonstrated that neutrophils isolated from HCs underwent spontaneous apoptosis following a 6 and 18 h ex vivo culture, with the induction of cell death accelerated by treatment with the transcription inhibitor Act D or the protein synthesis inhibitor CHX (Figure 1A, Supplementary Figure S6). At the molecular level, CHX treatment resulted in a rapid and significant reduction in the expression of the anti-apoptotic protein Mcl-1 (Figure 1B).

Neutrophils isolated from trauma patients ≤ 1, 4–12 and 48–72 h post-injury underwent significantly less spontaneous apoptosis during a 6 (*p* < 0.005) and/or 18 h (*p* < 0.05) ex vivo culture when compared to the results for neutrophils from HCs (Figure 1C). Furthermore, in our accelerated models of apoptosis, a significantly greater percentage of neutrophils isolated from trauma patients 4–12 and 48–72 h post-injury exhibited an Annexin V^−^ PI^−^ phenotype following a 6 h treatment with Act D or CHX (Figure 1D). In our 18 h ex vivo cultures, a positive association was observed between the percentage of alive neutrophils and patient ISS at our pre-hospital (r(n = 10) = 0.808, *p* = 0.0047) and 48–72 h (r(n = 15) = 0.619, *p* = 0.0138) sampling timepoints. Similarly, 48–72 h post-injury, the percentage of Annexin V^−^ PI^−^ neutrophils recovered from cultures treated with CHX or Act D positively correlated with the ISS (CHX, r(n = 14) = 0.654, *p* = 0.011; Act D, (r(n = 14) = 0.716, *p* = 0.004).

### 3.3. Traumatic Injury Results in Delayed Turnover of Mcl-1, Reduced Mitochondrial Membrane Depolarisation, and Decreased Activation of Caspase 3

**Figure 2:**
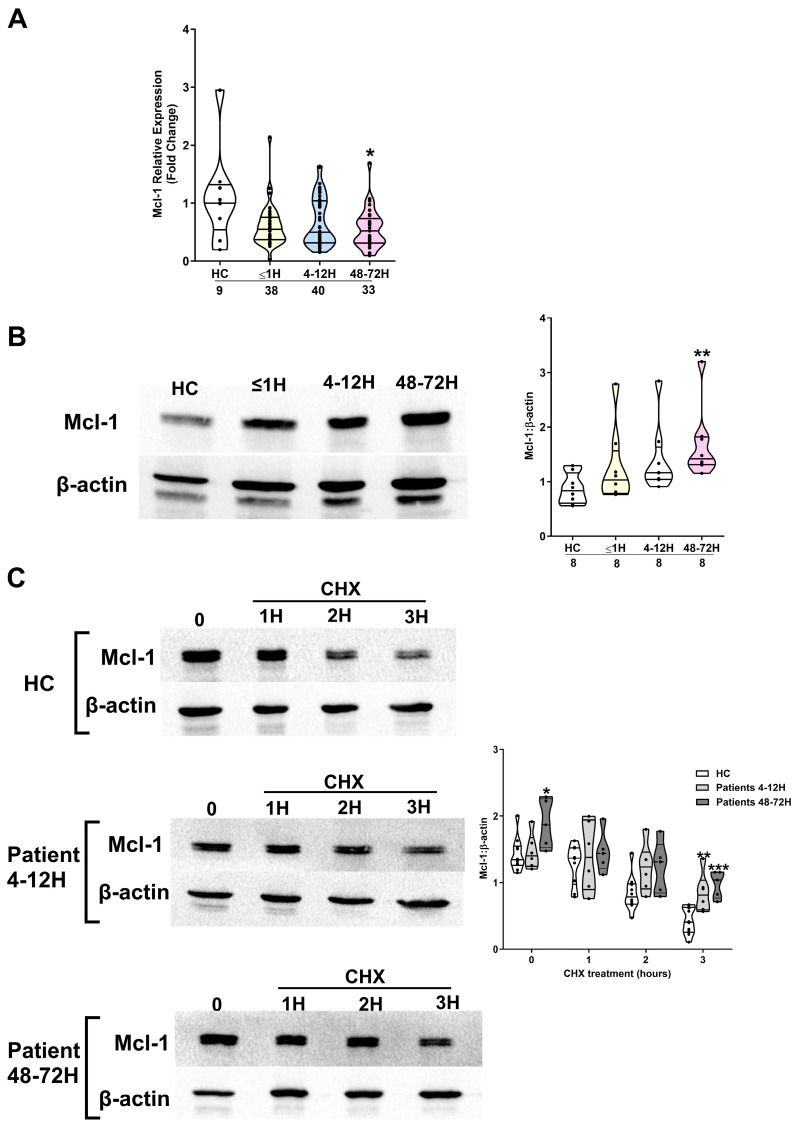
Traumatic injury promotes stabilisation of the anti-apoptotic protein Mcl-1. (**A**,**B**) Expression of Mcl-1 at the mRNA (**A**) and protein (**B**) level in freshly isolated neutrophils obtained from HCs and trauma patients at three post-injury timepoints (≤1, 4–12, and 48–72 h). The number of samples analysed is indicated below each timepoint. * *p* < 0.05, ** *p* < 0.005 vs. HCs. In (**B**), a representative Western blot is presented in the left panel, with collated densitometry data (n = 8) presented in the right panel. (**C**) Mcl-1 protein expression in neutrophils isolated from HCs (n = 10; top panel) and trauma patients 4–12 (n = 6; middle panel) and 48–72 (n = 5; bottom panel) hours post-injury following a 1, 2, and 3 h ex vivo culture in the presence of the protein synthesis inhibitor CHX (50 µM). A representative Western blot is shown for HCs and patients, alongside collated densitometry data. * *p* < 0.05, ** *p* < 0.005, *** *p* < 0.0005 vs. HCs.

A comparison of Mcl-1 gene and protein expression in freshly isolated neutrophils from HCs and trauma patients revealed a significant injury-induced reduction in Mcl-1 mRNA levels and increased Mcl-1 protein expression at the 48–72 h post-injury sampling timepoint (*p* < 0.05; Figure 2A,B).

To determine whether traumatic injury prolonged the half-life of Mcl-1, we treated neutrophils isolated from trauma patients and HCs for 1, 2, and 3 h with CHX before measuring the expression of this anti-apoptotic protein. As shown in Figure 2C, in the presence of this protein synthesis inhibitor, Mcl-1 levels remained significantly higher in neutrophils obtained from trauma patients 4–12 and 48–72 h post-injury when compared to the results for HCs following a 3 h CHX treatment.

**Figure 3:**
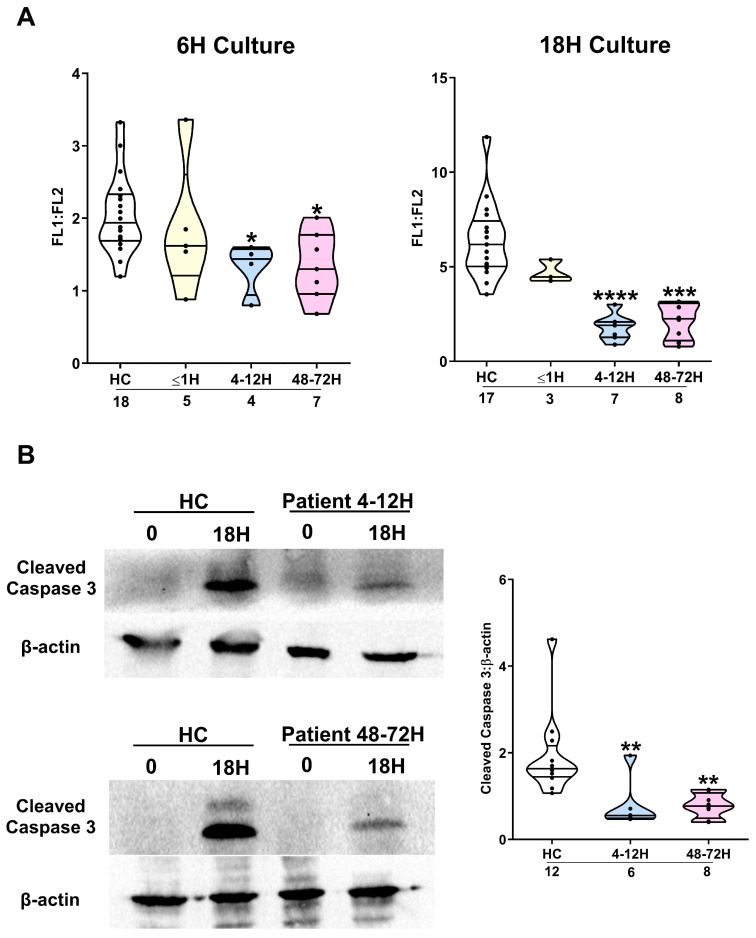
Traumatic injury results of delayed activation of the intrinsic apoptosis pathway in neutrophils. (**A**) Compared to HCs, neutrophils isolated from trauma patients 4–12 and 48–72 h post-injury exhibited reduced mitochondrial membrane depolarisation following a 6 h (left panel) or 18 h (right panel) ex vivo culture. The number of samples analysed is indicated below each timepoint. * *p* < 0.05, *** *p* < 0.0005, **** *p* < 0.0001 vs. HCs. (**B**) Comparison of active caspase-3 protein expression in neutrophils isolated from HCs and trauma patients at two post-injury timepoints (4–12 and 48–72 h) following an 18 h ex vivo culture. Data are presented as representative Westerns blots (left panel) and collated densitometry data (right panel). The number of samples analysed is indicated below each timepoint. ** *p* < 0.005 vs. HCs.

Mcl-1 confers apoptotic resistance by promoting stabilisation of mitochondrial membrane potential. We found that neutrophils isolated from trauma patients 4–12 and 48–72 h post-injury exhibited a significantly reduced level of mitochondrial membrane depolarisation following a 6 or 18 h ex vivo culture when compared to the results for the HCs (Figure 3A). This retention in mitochondrial membrane potential was accompanied by delayed activation of caspase-3 (Figure 3B).

Screening of neutrophil lysates from HCs and trauma patients for other Bcl-2 family members revealed that traumatic injury had no effect upon the expression of the anti-apoptotic protein A1 or the pro-apoptotic protein Bax (Supplementary Figure S7A,B).

### 3.4. Effect of Serum Treatment on Spontaneous Neutrophil Apoptosis

**Figure 4:**
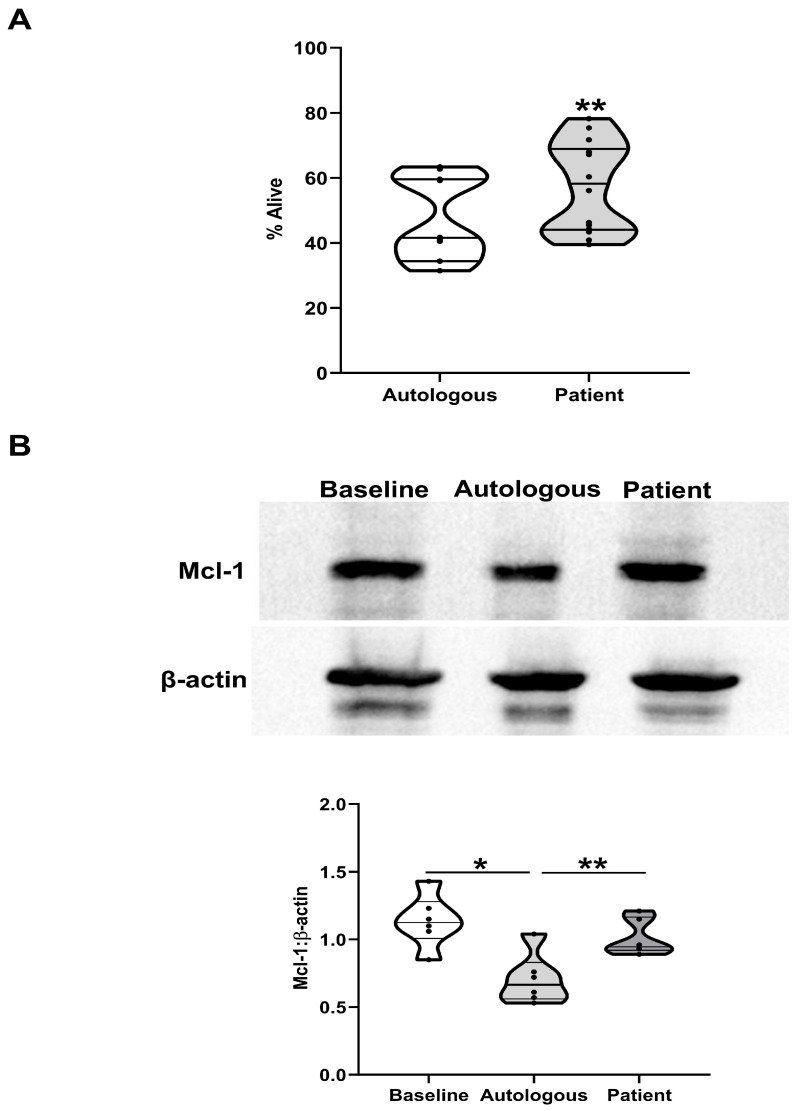
Effect of patient serum on neutrophil spontaneous apoptosis and Mcl-1 protein expression. (**A**) Neutrophils isolated from HCs were incubated for 18 h in media supplemented with 10% autologous serum or serum obtained from trauma patients 4–12 h post-injury (n = 15). Post-incubation, the percentage of Annexin V^−^/PI^−^ neutrophils was measured. ** *p* < 0.005 vs. autologous serum. (**B**) Mcl-1 protein expression, measured by Western blotting, in HCs neutrophils immediately post-isolation (baseline) or following a 6 h culture in media supplemented with 10% autologous serum or serum obtained from trauma patients 4–12 h post-injury. Data are presented as a representative Western blot (top panel) and collated densitometry data (n = 5, bottom panel). * *p* < 0.05, ** *p* < 0.005.

To determine whether factors released into the circulation post-injury can promote neutrophil survival, neutrophils isolated from HCs were cultured for 18 h in media supplemented with 10% autologous serum or serum obtained from trauma patients 4–12 h post-injury. As shown in Figure 4A, a significantly greater percentage of Annexin V^−^/PI^−^ non-apoptotic neutrophils was recovered from cultures that were treated with trauma patient sera (*p* < 0.005). This serum-induced extension of lifespan was accompanied by delayed turnover of Mcl-1 (Figure 4B).

### 3.5. Effect of MtDAMP Treatment on Neutrophil Spontaneous Apoptosis and Anti-Microbial Activity

**Figure 5:**
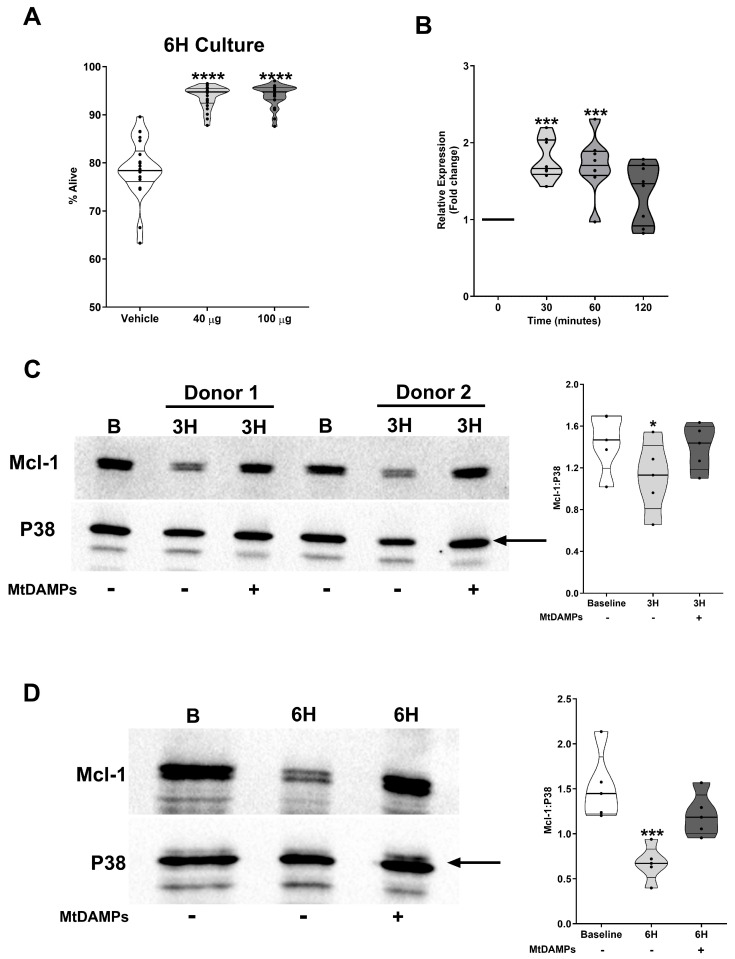
Exposure to mtDAMPs prolongs neutrophil lifespan in vitro. (**A**) Neutrophils isolated from HCs (n = 22) were cultured ex vivo for 6 h in the presence of 40 or 100 µg/mL mtDAMPs or vehicle control (PBS), after which the percentage of alive neutrophils, defined as Annexin V^−^/PI^−^, was measured. **** *p* < 0.0001 vs. vehicle. (**B**) Mcl-1 mRNA levels in neutrophils isolated from HCs (n = 8) following a 0–2 h stimulation with 40 µg/mL mtDAMPs. *** *p* < 0.0005 vs. 0. (**C**,**D**) Comparison of Mcl-1 protein expression measured in lysates prepared from HCs neutrophils immediately post-isolation (baseline) and following a (**C**) 3 h or (**D**) 6 h culture in the presence of 40 µg/mL mtDAMPs or vehicle control (PBS, n = 5). A representative Western blot is presented in the left panel, with collated densitometry data presented in the right panel. In (**C**) * *p* < 0.05 vs. baseline. In (**D**) *** *p* < 0.0005 vs. baseline. B, baseline. In (**C**,**D**), the arrow indicates the band of the loading control used in densitometry analysis.

**Figure 6:**
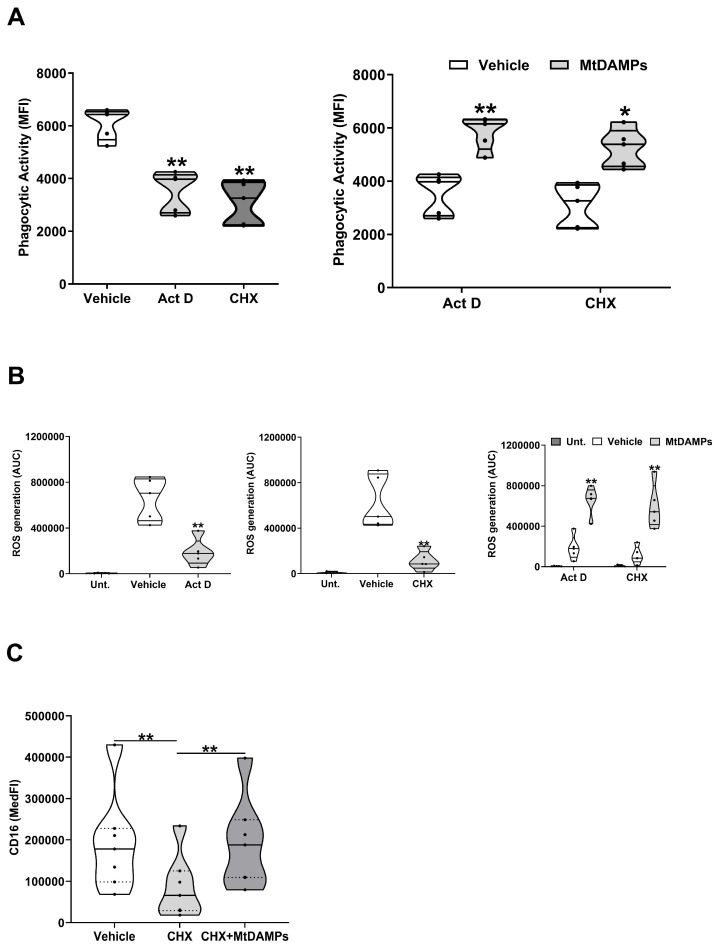
The mtDAMP-induced extension of neutrophil lifespan results in the retention of anti-microbial activity. (**A**) Left panel: phagocytic activity of neutrophils isolated from HCs (n = 5) following a 6 h pre-treatment with the transcription inhibitor Act D (1 µM), the protein synthesis inhibitor CHX (50 µM), or vehicle control (DMSO). Right panel: phagocytic activity of neutrophils isolated from HCs (n = 5) following a 6 h culture with 1 µM Act D or 50 µM CHX in the presence of 100 µg/mL mtDAMPs or vehicle control (DMSO and PBS). * *p* < 0.05, ** *p* < 0.005 vs. vehicle. (**B**) PMA-induced ROS production by neutrophils isolated from HCs (n = 5) following a 6 h pre-treatment with vehicle control (DMSO), Act D (1 µM, left panel), or CHX (50 µM, middle panel). Right panel: PMA-induced ROS production by neutrophils isolated from HCs (n = 5) following a 6 h culture with 1 µM Act D or 50 µM CHX in the presence of 100 µg/mL mtDAMPs or vehicle control (DMSO and PBS). ** *p* < 0.005 vs. vehicle. (**C**) CD16 expression on the surface of neutrophils treated for 6 h with vehicle control (DMSO and PBS), 50 µM CHX, or 50 µM CHX and 100 µg/mL mtDAMPs (n = 7). ** *p* < 0.005. AUC, area under the curve; MFI, mean fluorescence intensity; MedFI, median fluorescence intensity.

To determine whether exposure to mtDAMPs extended neutrophil lifespan, neutrophils isolated from HCs were treated for 6 h with 40 or 100 µg/mL mtDAMPs, after which apoptosis was assessed via Annexin V and PI staining. Treatment with either dose of mtDAMPs resulted in the recovery of a significantly higher percentage of Annexin V^−^/PI^−^ non-apoptotic neutrophils when compared to the results for the vehicle-treated controls (Figure 5A). Ruling out a role for endotoxin contamination in our mtDAMP preparations, the pre-treatment of neutrophils with polymyxin B did not prevent the mtDAMP-induced delay in neutrophil apoptosis (Supplementary Figure S8). At the molecular level, the extension of neutrophil lifespan observed with mtDAMP treatment occurred alongside a significant increase in Mcl-1 mRNA levels (Figure 5B) and preservation of Mcl-1 protein expression following a 3 h (Figure 5C) or 6 h culture (Figure 5D).

To determine whether the delay in neutrophil apoptosis conferred by mtDAMPs resulted in the retention of cellular function, we compared the anti-microbial activities of HCs neutrophils treated for 6 h with Act D or CHX in the presence or absence of mtDAMPs. Exposure to Act D or CHX alone suppressed neutrophil phagocytic activity and ROS generation (Figure 6A,B). The addition of mtDAMPs counteracted both the Act D and CHX-induced impairment of neutrophil anti-microbial activity (Figure 6A,B). Expression of the Fc receptor CD16 on the surface of the neutrophils is critical for their efficient uptake of opsonised pathogens [39]. Compared to vehicle controls, CHX-treated neutrophils from HCs exhibited significantly reduced CD16 surface density (*p* < 0.005; Figure 6C). In contrast, we found that CD16 expression on neutrophils co-cultured with CHX and mtDAMPs was significantly higher than that recorded for the CHX-only treated cells and was not significantly different from the surface density of CD16 recorded for the vehicle-treated controls (*p* < 0.005; Figure 6C).

### 3.6. Exposure to MtDAMPs Delays the Turnover of Mcl-1

**Figure 7:**
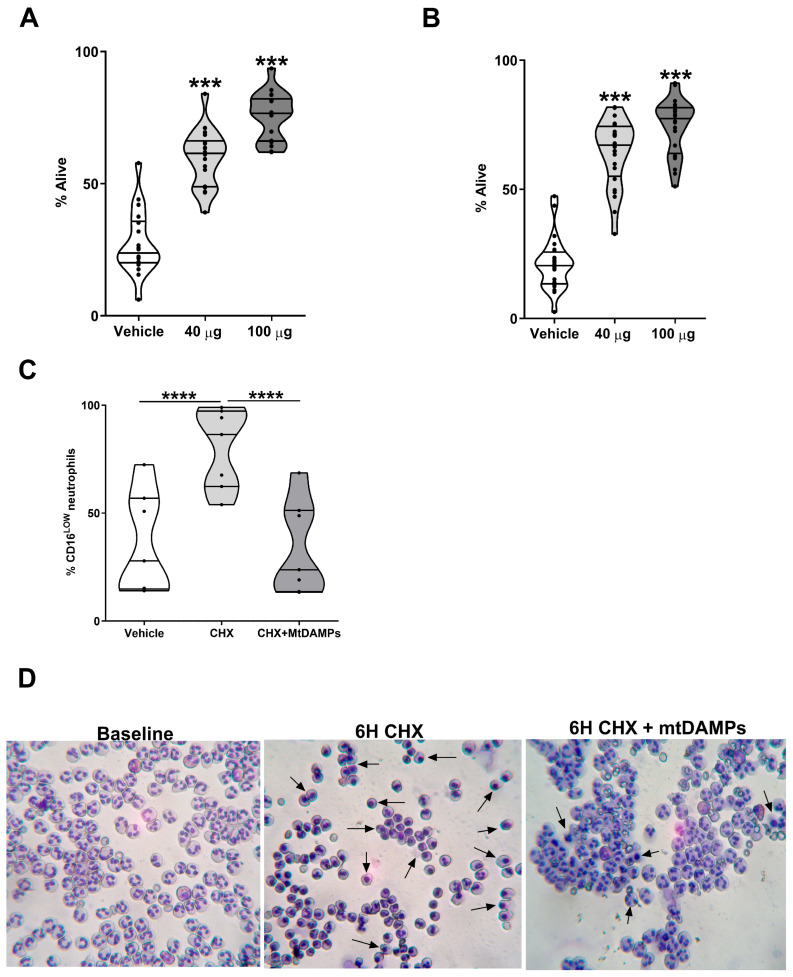
MtDAMPs prolong neutrophil half-life in a protein synthesis-independent manner. (**A**,**B**) Neutrophils isolated from HCs (n = 18–24) were cultured for 6 h with 1 µM Act D (**A**) or 50 µM CHX (**B**) in the presence or absence of 40 or 100 µg/mL mtDAMPs, after which the percentage of alive (Annexin V^−^/PI^−^) cells were determined. *** *p* < 0.0005 vs. vehicle. Vehicle denotes neutrophils co-treated for 6 h with DMSO and PBS, the vehicle controls for Act D/CHX, and mtDAMPs, respectively. (**C**) Percentage of CD16^LOW^ apoptotic neutrophils recovered from cell cultures treated for 6 h with vehicle control (DMSO and PBS), 50 µM CHX, or 50 µM CHX and 100 µg/mL mtDAMPs. Data were acquired from seven independent experiments analysing neutrophils from HCs. **** *p* < 0.0001 vs. vehicle. (**D**) Light microscope images showing the morphology of neutrophils immediately post-isolation (baseline) and following a 6 h treatment with 50 µM CHX in the presence or absence of 100 µg/mL mtDAMPs. Arrows indicate apoptotic neutrophils. Data are representative of five independent experiments.

To investigate whether the mtDAMP-induced suppression of neutrophil apoptosis required de novo RNA and protein synthesis, we first performed Annexin V and PI staining on the HC neutrophils co-treated for 6 h with mtDAMPs and either Act D or CHX. At doses of 40 and 100 µg/mL, mtDAMPs protected neutrophils against both Act D and CHX-induced apoptosis (Figure 7A,B). Aside from externalisation of phosphatidylserine, reduced surface expression of CD16 is a reported characteristic of apoptotic neutrophils [40]. Confirming this, we detected, in cell cultures treated with CHX, a significantly higher percentage of Annexin V^+^ neutrophils within the CD16^LOW^ fraction when compared to that in the CD16^HIGH^ neutrophils (Supplementary Figure S9). As expected, compared to the vehicle-treated controls, a significantly higher frequency of CD16^LOW^ neutrophils were recovered from cell cultures treated with CHX for 6 h (Figure 7C). The percentage of CD16^LOW^ neutrophils detected in CHX and mtDAMP co-treated cultures was comparable to that of the vehicle control and significantly lower than the frequency recovered from the CHX-only treated cultures (*p* < 0.0001; Figure 7C). Confirming our flow cytometric data, morphological assessment of neutrophils from HCs demonstrated the anti-apoptotic properties of mtDAMPs. As shown in Figure 7D, following a 6 h treatment, CHX-treated neutrophils exhibited clumping, a reduction in cell volume, and chromatin condensation, three characteristics of apoptosed cells. These features were less apparent for neutrophils co-cultured with 100 µg/mL mtDAMPs and CHX (Figure 7D).

**Figure 8:**
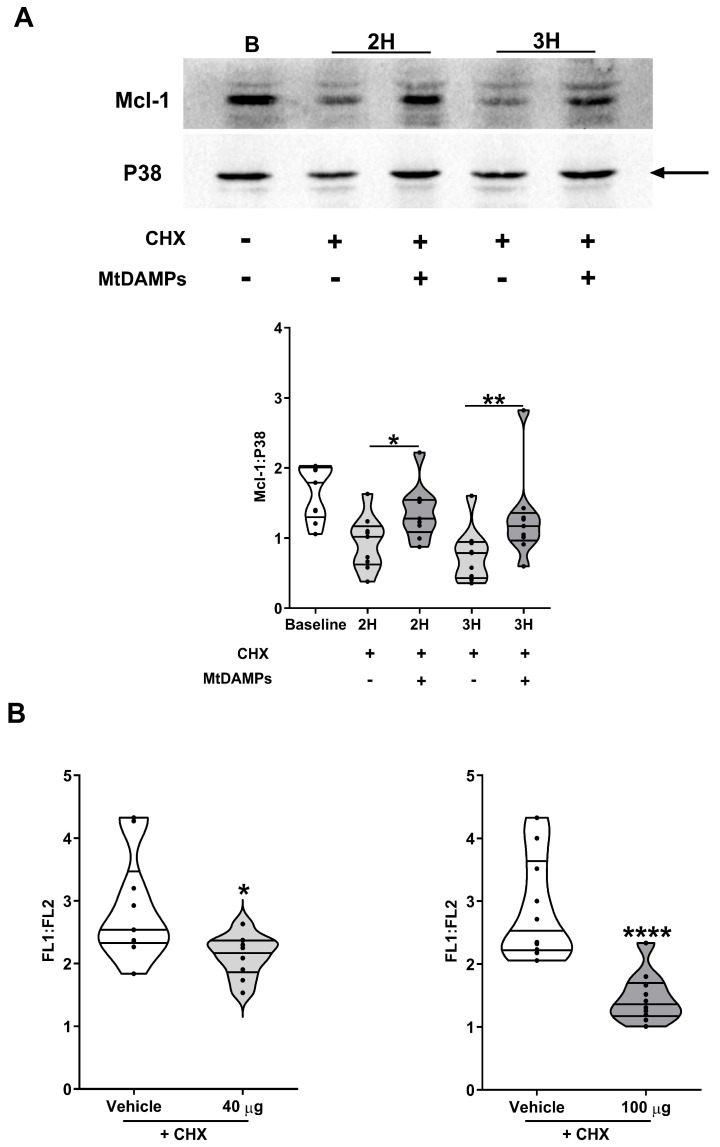
Exposure to mtDAMPs delays CHX-induced Mcl-1 turnover and mitochondrial membrane depolarisation. (**A**) Mcl-1 protein expression in neutrophils from HCs immediately post-isolation (baseline) or following a 2 or 3 h treatment with 50 µM CHX in the presence or absence of 40 µg/mL mtDAMPs (n = 9). * *p* < 0.05, ** *p* < 0.005. Data are presented as a representative Western blot (top panel) and as collated densitometry data (bottom panel). The arrow indicates the band of the loading control used in densitometry analysis. (**B**) Comparison of mitochondrial transmembrane potential in neutrophils from HCs treated for 6 h with 50 µM CHX in the presence or absence of 40 µg/mL (left panel) or 100 µg/mL (right panel) mtDAMPs. Data from 10 independent experiments are presented. * *p* < 0.05, **** *p* < 0.0001 vs. vehicle.

The resistance to CHX-induced apoptosis conferred by mtDAMP treatment led us to hypothesise that mtDAMPs delayed the turnover of the anti-apoptotic protein Mcl-1. Compared to non mtDAMP treated neutrophils, we detected significantly higher Mcl-1 protein expression in CHX-treated neutrophils from HCs following a 2 or 3 h co-incubation with mtDAMPs (Figure 8A). This stabilisation of Mcl-1 occurred alongside a significant delay in the loss of mitochondrial transmembrane potential that was observed in vehicle-treated neutrophils from HCs (Figure 8B). Exposure to mtDAMPs had no effect upon the expression of the pro-apoptotic protein Bax in CHX-treated neutrophils of the HCs (Supplementary Figure S10).

### 3.7. MtDAMP Treatment Delays CHX-Induced Activation of Caspase-3 That Promotes the Turnover of Mcl-1

**Figure 9:**
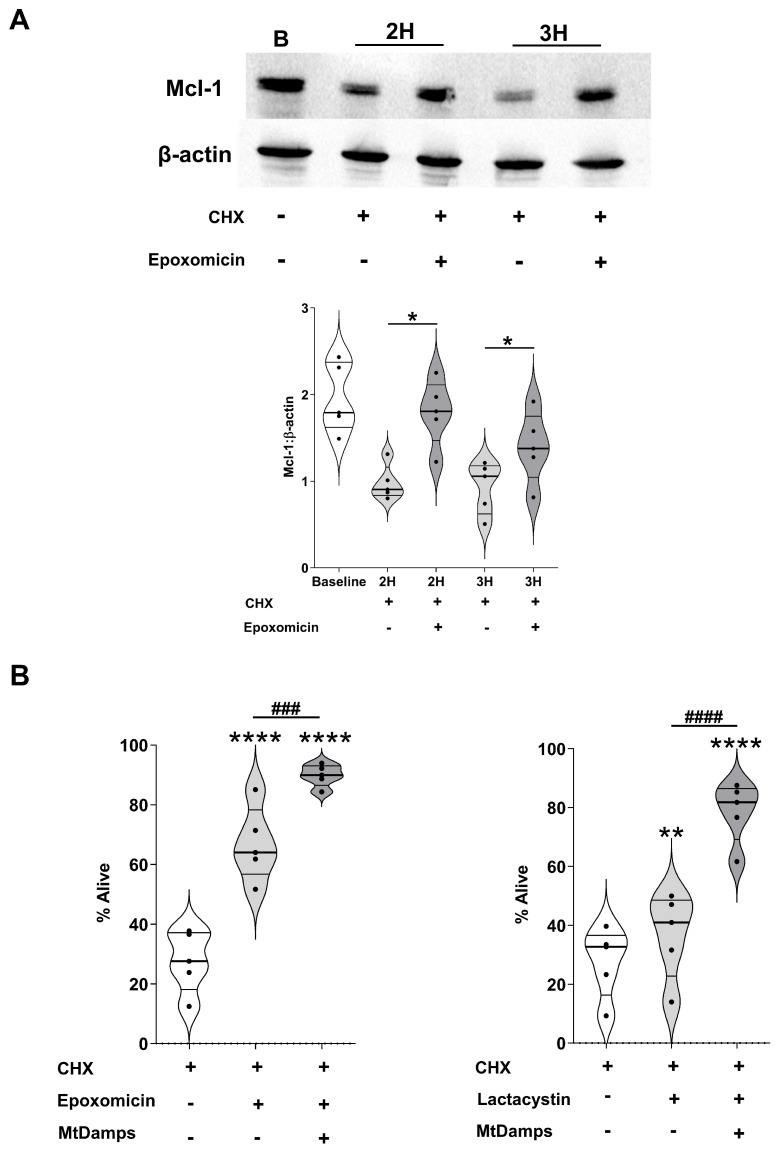
Inhibition of the proteasome delays the turnover of Mcl-1 and prolongs neutrophil life-span. (**A**) Mcl-1 protein expression in freshly isolated neutrophils (baseline) and in neutrophils treated for 1 h with the proteasome inhibitor epoxomicin or vehicle control (DMSO) prior to a 2 or 3 h culture with the protein synthesis inhibitor CHX (50 µM). Data obtained from 5 HCs are presented as a representative Western blot (top panel) or as collated densitometry data (bottom panel). * *p* < 0.05. (**B**) Following a 1 h pre-treatment with the proteasome inhibitors epoxomicin (left panel) or lactacystin (right panel), neutrophils isolated from HCs (n = 5) were cultured for 6 h with 50 µM CHX in the presence or absence of 100 µg/mL mtDAMPs, after which neutrophil apoptosis was assessed by annexin V/PI staining. Alive cells were defined as Annexin V^−^/PI^−^. ** *p* < 0.005, **** *p* < 0.0001 vs. CHX treatment only. ^###^ *p* < 0.0005; ^####^ *p* < 0.0001.

Degradation of Mcl-1 by the proteasome is a trigger for neutrophil apoptosis. As shown in Figure 9A, neutrophils from HCs pre-treated with the proteasome inhibitor epoxomicin exhibited significantly higher Mcl-1 protein expression following a 2 or 3 h treatment with CHX when compared to neutrophils pre-treated with the vehicle control. A concurrent assessment of apoptosis revealed a greater recovery of Annexin V^−^/PI^−^ non-apoptotic neutrophils from cultures co-treated with epoxomicin and CHX when compared to the results for CHX alone (Figure 9B). This resistance to CHX-induced apoptosis was also observed in neutrophils treated with lactacystin, an alternative inhibitor of the proteasome (Figure 9B).

We hypothesised that if the sole mechanism by which mtDAMPs prolonged neutrophil lifespan was the delay of the proteasome-mediated turnover of Mcl-1, then a combined treatment of mtDAMPs and proteasome inhibitors would have no additional effect on neutrophil lifespan than that obtained with either treatment alone. Compared to cultures treated with epoxomicin or lactacystin, we recovered a significantly higher frequency of Annexin V^−^/PI^−^ non-apoptotic neutrophils from cultures co-treated with mtDAMPs and either of these proteasome inhibitors (Figure 9B).

**Figure 10:**
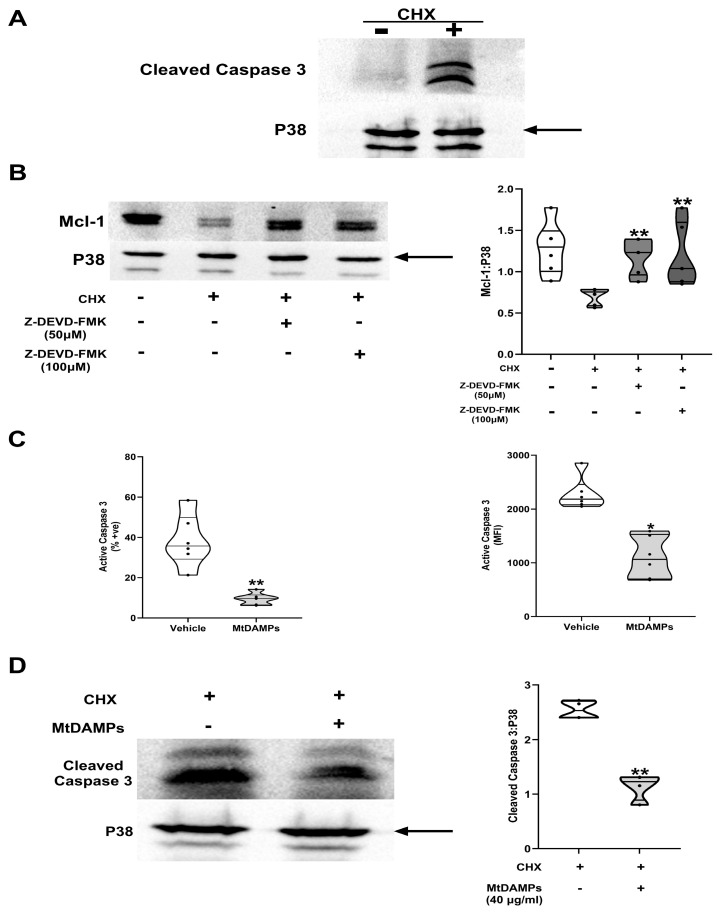
MtDAMP treatment delays CHX-induced activation of caspase-3 that promotes the turnover of Mcl-1. (**A**) Representative Western blot depicting cleaved caspase-3 expression in neutrophils from HCs (n = 3) immediately post-isolation (baseline) and following a 3 h treatment with the protein synthesis inhibitor CHX (50 µM). The arrow indicates the band of the loading control used in densitometry analysis. (**B**) Mcl-1 protein expression in neutrophils treated with the caspase-3 inhibitor Z-DEVD-FMK (50 or 100 µM) or vehicle control (DMSO) for 1 h prior to a 3 h culture with 50 µM CHX. Data are presented as a representative Western blot (left panel) and as collated densitometry data from six independent experiments. Neutrophils were isolated from HCs. ** *p* < 0.005 vs. CHX. The arrow indicates the band of the loading control used in densitometry analysis. (**C**) Active caspase-3 expression in neutrophils isolated from HCs (n = 6) following a 6 h treatment with 50 µM CHX in the presence of 100 µg/mL mtDAMPs or vehicle (PBS). Data are presented as the percentage of neutrophils expressing active caspase-3 (left panel) and active caspase-3 mean fluorescence intensity (MFI) values. * *p* < 0.05, ** *p* < 0.005 vs. vehicle. (**D**) Representative Western blot (left panel) and collated densitometry data (right panel, n = 4) for cleaved caspase-3 in HCs neutrophils treated for 3 h with 50 µM CHX in the presence of 40 µg/mL mtDAMPs or vehicle (PBS). ** *p* < 0.005 vs. CHX treatment only. The arrow indicates the band of the loading control used in densitometry analysis.

In addition to the proteasome, caspase-3 promotes neutrophil apoptosis by cleaving Mcl-1 [41]. In our model of accelerated apoptosis, we detected the activation of caspase-3 in HC neutrophils subjected to a 3 h CHX treatment (Figure 10A). Pre-treating neutrophils with the selective caspase-3 inhibitor Z-DEVD-FMK at concentrations of 50 or 100 µM delayed this CHX-induced activation of caspase-3 (Supplementary Figure S11). Demonstrating a role for caspase-3 in modulating Mcl-1 expression under this treatment condition, we found that levels of this anti-apoptotic protein were significantly higher in CHX-exposed neutrophils from HCs that had been pre-treated with Z-DEVD-FMK when compared to the levels for the vehicle control (Figure 10B).

Based on these results, we proposed that mtDAMP treatment may promote Mcl-1 stabilisation by delaying the activation of caspase-3. As shown in Figure 10C, flow cytometric analysis of HCs neutrophils revealed significantly lower caspase-3 activation in neutrophils co-treated with CHX and mtDAMPs when compared to CHX- and vehicle-treated cultures. This finding was confirmed by Western blotting, where co-culture with mtDAMPs significantly reduced the cleavage of caspase-3 that was observed following a 3 h treatment with CHX alone (Figure 10D).

### 3.8. Reduced Mitochondrial Membrane Depolarisation and Delayed Turnover of Mcl-1 Are Features of MtDNA-Induced Extension of Neutrophil Lifespan

**Figure 11:**
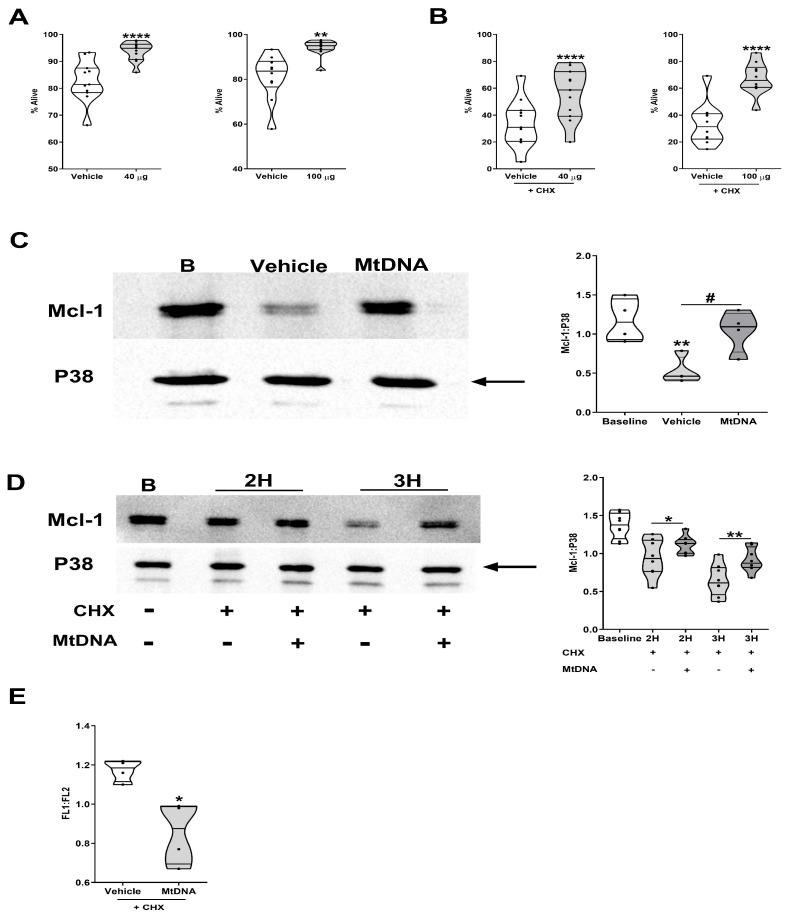
Treatment with purified mtDNA extends neutrophil life-span in vitro. (**A**) Following a 6 h culture with 40 µg/mL (left panel, n = 11), 100 µg/mL (right panel, n = 10) mtDNA, or vehicle control (PBS), the percentage of alive (Annexin V^−^/PI^−^) neutrophils isolated from HCs was determined by flow cytometry. ** *p* < 0.005, **** *p* < 0.0001 vs. vehicle. (**B**) The percentage of alive (Annexin V^−^/PI^−^) neutrophils recovered from cultures treated for 6 h with 50 µM CHX in the presence of 40 µg/mL (left panel, n = 11), 100 µg/mL (right panel, n = 10) mtDNA, or vehicle control. **** *p* < 0.0001 vs. vehicle. (**C**) Mcl-1 protein expression in neutrophils isolated from HCs immediately post-isolation (baseline, B) or following a 6 h treatment with 40 µg/mL mtDNA or vehicle (PBS). Data are presented as a representative Western blot (left panel) and as collated densitometry data (right panel, n = 4). ** *p* < 0.005 vs. baseline; # *p* < 0.05. (**D**) Mcl-1 protein expression in neutrophils isolated from HCs (n = 8) immediately post-isolation (baseline, B) or following a 2 or 3 h treatment with 50 µM CHX in the presence of 40 µg/mL mtDNA or vehicle (PBS). Data are presented as a representative Western blot (left panel) and as collated densitometry data (right panel). * *p* < 0.05; ** *p* < 0.005. In (**C**,**D**), the arrow indicates the band of the loading control used in densitometry analysis. (**E**) Comparison of mitochondrial transmembrane potential in neutrophils isolated from HCs (n = 4) following a 6 h culture with 50 µM CHX in the presence of 40 µg/mL mtDNA or vehicle control (PBS). * *p* < 0.05 vs. vehicle.

MtDAMPs are a heterogeneous collection of proteins, lipids, and DNA, with mitochondrial-derived N-formylated peptides and mtDNA being two potent modulators of neutrophil function [38,42,43,44]. Demonstrating an anti-apoptotic effect of mtDNA, we detected, in our spontaneous and CHX-accelerated models of apoptosis in HC neutrophils, a significantly greater percentage of neutrophils with an Annexin V^−^/PI^−^ phenotype following a 6 h treatment with 40 or 100 µg/mL purified mtDNA when compared to the results for the vehicle control (Figure 11A,B). This mtDNA-induced increase in lifespan, which remained when the HC neutrophils were pre-treated with polymyxin B (Supplementary Figure S12) and was accompanied by a delayed turnover of Mcl-1 (Figure 11C,D) and a reduced depolarisation of mitochondrial transmembrane potential (Figure 11E).

**Figure 12:**
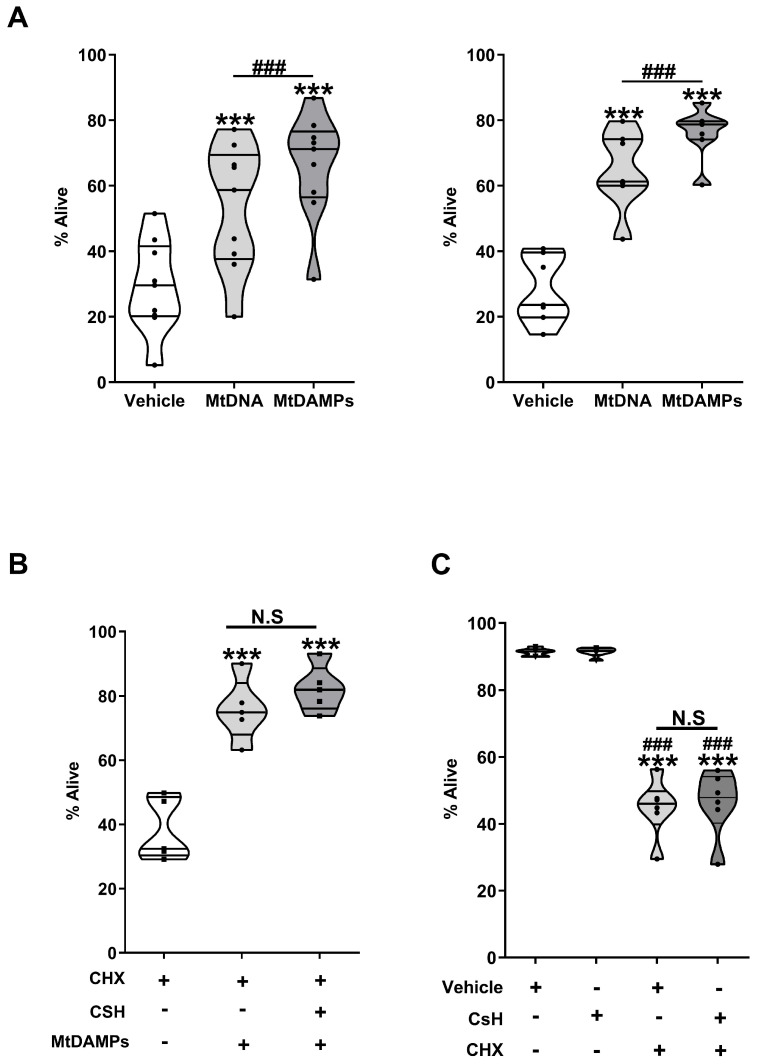
Exposure to mitochondrial-derived N-formylated peptides does not alter neutrophil life-span in vitro. (**A**) Frequency of alive (Annexin V^−^/PI^−^) neutrophils in cultures treated for 6 h with 50 µM CHX in the presence of either 40 µg/mL (left panel, n = 9) or 100 µg/mL (right panel, n = 7) mtDAMPs, purified mtDNA, or vehicle control (PBS). *** *p* < 0.0005 vs. vehicle; ^###^ *p* < 0.0005. (**B**) Following a 1 h pre-treatment with 1 µM CsH or vehicle control (DMSO), neutrophils from HCs were treated for 6 h with 50 µM CHX and 100 µg/mL mtDAMPs, after which the percentage of alive (Annexin V^−^/PI^−^) cells was determined by flow cytometry (n = 5); *** *p* < 0.0005 vs. CHX treatment. N.S., non-significant. (**C**) Following a 1 h pre-treatment with 1 µM CsH or vehicle control (DMSO), neutrophils from HCs were treated for 6 h with 50 µM CHX, after which the percentage of alive (Annexin V^−^/PI^−^) cells was determined by flow cytometry (n = 6). *** *p* < 0.0005 vs. vehicle; ^###^ *p* < 0.0005 vs. CsH.

A comparison of CHX-induced apoptosis rates between neutrophils isolated from HCs and treated with purified mtDNA and whole mtDAMP preparations revealed that a significantly higher frequency of Annexin V^−/^PI^−^ non-apoptotic cells were present in cultures treated with mtDAMPs (*p* < 0.0005; Figure 12A). Suggesting that other mtDAMPs, in addition to mtDNA, possess anti-apoptotic properties, we investigated whether mitochondrial-derived N-formylated peptides could prolong neutrophil life-span by treating neutrophils with the selective FPR-1 antagonist CsH prior to mtDAMP treatment. As shown in Figure 12B and Supplementary Figure S13, we found no difference in the percentage of Annexin V^−^/PI^−^ non-apoptotic neutrophils between vehicle and CsH pre-treated cells following a 6 h co-culture with 100 µg/mL mtDAMPs and CHX. CsH treatment alone had no effect upon neutrophil viability when compared to that of the vehicle, nor did it rescue CHX-induced apoptosis (Figure 12C and Supplementary Figure S14).

## 4. Discussion

A hyperactive pool of circulating neutrophils that exhibit enhanced basal migration, ROS production, and degranulation is a feature of the systemic inflammatory response syndrome triggered by major traumatic and thermal injury [7,8,9]. Exacerbating this state of dysfunction is an extension of neutrophil lifespan. This post-injury delay in neutrophil apoptosis has been linked to the development of secondary complications such as MODS and MOF [15,18]. Although associations have been reported between the inhibition of neutrophil apoptosis and poor clinical outcomes, few studies have addressed the mechanisms that underpin the prolonged lifespan of neutrophils post-injury and the circulating factors that confer apoptotic resistance.

In line with previous results generated from the analysis of post-hospital admission blood samples [17,18,20,21], we found that neutrophils isolated from trauma patients 4–12 and 48–72 h post-injury exhibited delayed spontaneous apoptosis following 6 and 18 h ex vivo culture. Adding to these observations, we report, for the first time, that this trauma-induced resistance to apoptosis is evident during the ultra-early inflammatory response to injury, with an increased frequency of neutrophils isolated from trauma patients within 1 h of injury exhibiting an Annexin V^−^/PI^−^ phenotype following an 18 h culture when compared to neutrophils from HCs. Pointing towards injury severity as a factor that influences neutrophil lifespan, a positive association was found between the percentage of live neutrophils recovered from 18 h ex vivo cultures and a patient’s ISS. This observation is similar to data reported by Nolan et al. who found a negative association between neutrophil apoptosis rates and a patient’s APACHE score, a classification system that is used to measure the severity of disease in ICU patients [17].

Key events that precede the induction of neutrophil spontaneous apoptosis are the loss of mitochondrial transmembrane potential and activation of the executioner caspase, caspase-3. Mirroring the results reported in a rodent model of severe thermal injury [30] and offering a potential mechanistic explanation for the trauma-induced extension of neutrophil lifespan, we found that neutrophils isolated from trauma patients 4–12 and 48–72 h post-injury exhibited a significantly reduced level of mitochondrial membrane depolarisation following a 6 and 18 h ex vivo culture. As expected, this retention in mitochondrial membrane potential occurred alongside the delayed activation of caspase-3. At the molecular level, mitochondrial membrane potential is regulated by pro- and anti-apoptotic proteins of the Bcl-2 family.

Previous studies have attributed the post-trauma extension of neutrophil half-life to increased expression of the anti-apoptotic protein Mcl-1 and reduced expression of the pro-apoptotic protein Bax [21,30,45]. Our analysis of neutrophil lysates prepared immediately post-isolation revealed a significant upregulation in the expression of Mcl-1. As an anti-apoptotic protein whose intracellular levels correlate positively with neutrophil lifespan [21,45,46], it was notable that despite observing a resistance of neutrophils to apoptosis at all three of our sampling timepoints, we detected an increased expression of Mcl-1 only in freshly isolated neutrophils acquired from patients 48–72 h post-injury. Therefore, this suggests that Mcl-1-independent mechanisms are also involved in promoting the trauma-induced extension of neutrophil lifespan reported here. Our data showing comparable expression of Bax and A1 in HCs and trauma patient neutrophils would appear to rule out a role for these molecules in delaying neutrophil apoptosis post-injury. Trauma-induced changes in the expression of other pro- and anti-apoptotic Bcl2 family members such as BCL-2, BCL-XL, BAK, and BID are possible explanations for increased neutrophil half-life and should be the focus of future studies that examine additional molecular mechanisms behind the modulation of neutrophil apoptosis following major injury.

As a short-lived protein with an estimated half-life of 1–5 h, we hypothesised that a post-injury delay in Mcl-1 turnover, rather than increased de novo synthesis, may contribute to the extended lifespan of neutrophils isolated from trauma patients. In line with this theory, we report, for the first time, that the neutrophils of trauma patients exhibit significantly increased expression of Mcl-1 following treatment with the protein synthesis inhibitor CHX. This enhanced stability of Mcl-1 was accompanied by increased survival rates of neutrophils in ex vivo cultures, an observation that corroborates results generated in a rodent model of thermal injury, where neutrophils isolated from burn-injured rats also displayed enhanced resistance to CHX-induced apoptosis [47].

In the absence of ex vivo stimulation, our data, showing delayed Mcl-1 turnover and apoptotic resistance of trauma patient neutrophils treated with transcription and translation inhibitors, pointing towards in vivo exposure to inflammatory agonists as being a signal that promotes neutrophil survival post-injury. In keeping with this theory, and in line with previous reports [15,16,17,21,22,31,32], we found that culturing neutrophils isolated from HCs in media supplemented with serum isolated from trauma patients significantly reduced their rate of spontaneous apoptosis and delayed the turnover of Mcl-1. It has previously been suggested that a post-injury elevation in circulating concentrations of GM-CSF contribute to these pro-survival effects, since neutralisation of this growth factor significantly reduced the ability of trauma patient sera to prolong the lifespan of HC neutrophils and preserve Mcl-1 expression during ex vivo culture [21,22]. However, in these studies, the neutralisation of GM-CSF did not totally eradicate the anti-apoptotic effects of patient sera, thereby implying the presence of additional pro-survival factors in the circulation of injured patients [21]. Results of studies that have attempted to identify such factors have thus far ruled out a role for interleukin (IL)-1, IL-6, tumour necrosis factor-alpha (TNF-α), and transforming growth factor-beta (TGF-β) [22].

Detected at elevated concentrations in the circulation of trauma patients in the minutes, hours, and days following injury [33,34,48], mtDAMPs are potent modulators of neutrophil function. Adding to a body of literature that has shown that mtDAMPs promote neutrophil migration, ROS generation, cytokine production, and degranulation [38,48], we have demonstrated here that mtDAMPs also deliver anti-apoptotic signals to the neutrophils. Compared to vehicle controls, neutrophils exposed to mtDAMPs exhibited significantly delayed rates of spontaneous apoptosis, as well as increased survival following treatment with transcription or translation inhibitors. This mtDAMP-induced delay in apoptosis resulted in a retention of cellular function, with neutrophils co-treated with mtDAMPs and either CHX or Act D exhibiting significantly increased phagocytic activity and ROS generation when compared to neutrophils treated with CHX or Act D alone. Phenotypic analysis revealed that mtDAMP treatment significantly reduced the CHX-induced loss of CD16 from the surface of neutrophils that occurred in the vehicle-treated controls. As an Fc receptor, whose expression correlates positively with the ability of neutrophils to phagocytose opsonised E.coli [39], this retention of CD16 offers a potential mechanistic explanation for the preservation of neutrophil phagocytic activity following mtDAMP exposure.

Previous studies have suggested that the preserved anti-microbial activity of apoptotic-resistant neutrophils would facilitate host defence [49]. However, these perceived benefits may not apply to the mtDAMP-induced delay in neutrophil apoptosis in the setting of traumatic injury. Indeed, characterised by enhanced basal activity and impaired functional responses to secondary challenge, neutrophil dysfunction is an immediate and persistent feature of the inflammatory response triggered by major trauma [2,4,6,7,8,9,10,11,12,13,14]. Thus, an mtDAMP-induced extension of neutrophil lifespan could conceivably contribute to increased longevity of a hyperactive and tolerant neutrophil pool that would promote tissue damage and organ injury, increasing patient susceptibility to nosocomial infections. Interestingly, it appears that mtDAMPs may directly contribute to both neutrophil dysfunction and increased lifespan post-trauma, with these outcomes driven by distinct mtDAMPs. For example, prior exposure to N-formylated peptides has been shown to significantly impair neutrophil migration and NET formation upon secondary stimulation [33,42], whilst the work of Bhagirath et al. [37] and the data we have presented here has demonstrated a role for mtDNA in delaying the induction of spontaneous and CHX-induced neutrophil apoptosis. Regarding the potential clinical relevance of these observations, a series of studies have reported that trauma patients who present with significantly elevated circulating concentrations of mtDNA are at increased risk of poor outcomes, such as mortality and the development of MOF and acute respiratory distress syndrome [50,51,52]. Alongside the immune-activating properties of mtDNA [43,44], our data, and that of others [37], would imply that increased concentrations of mtDNA may exacerbate inflammatory responses post-injury by promoting the survival of hyperactive and dysfunctional neutrophils.

Occurring alongside the delayed depolarisation of mitochondrial membrane potential, we found that the mtDAMP- and mtDNA-induced increase in neutrophil lifespan was independent of protein synthesis and coincided with the stabilisation of Mcl-1. Due to the presence of a PEST domain in its N-terminus, the turnover of Mcl-1 is regulated predominately by the proteasome. Interestingly, we found that whilst inhibition of the proteasome prevented the degradation of Mcl-1 in neutrophils treated with CHX for 2 h, the expression of this anti-apoptotic protein was significantly reduced, relative to baseline values, at 3 h post CHX treatment. With these data suggesting that an additional pathway was contributing to Mcl-1 turnover in CHX-treated neutrophils, we focused upon the activation of caspase-3, a cysteine protease, for which Mcl-1 is a known substrate [41]. Indeed, we found that pre-treating neutrophils with the caspase-3 inhibitor Z-DEVD-FMK significantly reduced the CHX-mediated decrease in Mcl-1 protein expression that was witnessed in the vehicle-treated controls. Suggesting that mtDAMP exposure may prolong Mcl-1 half-life by delaying the activation of caspase-3, we demonstrated, via flow cytometry and Western blotting, that the levels of cleaved caspase-3 were significantly lower in CHX-exposed neutrophils pre-treated with mtDAMPs when compared to the levels for the vehicle controls. Taken together, we suggest that the preservation of Mcl-1 expression in mtDAMP-treated neutrophils is attributable in part to delayed turnover by the proteasome and caspase-3.

Suggesting that other mtDAMPs, in addition to mtDNA, possess anti-apoptotic properties, we recovered a significantly higher frequency of Annexin V^−^/PI^−^ non-apoptotic neutrophils from cultures treated with whole mtDAMPs when compared to the results for purified mtDNA alone. Our experiments, which showed that prior treatment with the FPR-1 inhibitor CsH had no impact upon the ability of mtDAMPs to delay neutrophil apoptosis, combined with previous work demonstrating no effect of fMLP treatment on neutrophil survival [53], rule out a role for N-formylated peptides in contributing to the mtDAMP-induced extension of neutrophil lifespan. The existing literature has demonstrated that in vitro exposure to either adenosine triphosphate (ATP) or β-nicotinamide adenine dinucleotide (NAD^+^), two mitochondrial residing molecules, can inhibit neutrophil apoptosis by stabilising Mcl-1 expression, maintaining mitochondrial membrane potential, and delaying the activation of caspase-3, features reminiscent of what we observed in neutrophils treated with mtDAMPs [54,55]. Whilst no study, to our knowledge, has measured circulating levels of NAD^+^ post-injury, extracellular ATP levels have been reported to be elevated following both extracranial and traumatic brain injury [56,57,58]. Thus, we propose that the release of mtDNA and ATP from damaged tissue contributes, at least in part, to the post-injury extension of neutrophil lifespan.

## 5. Conclusions

We have shown that major traumatic injury results in an immediate and persistent delay in neutrophil apoptosis, which is accompanied by stabilisation of the anti-apoptotic protein Mcl-1, maintenance of mitochondrial membrane potential, and reduced activation of caspase-3. Moreover, we have demonstrated that this injury-induced extension of lifespan can be recapitulated in neutrophils isolated from HCs upon ex vivo exposure to mtDAMPs, thereby revealing anti-apoptotic effects for these alarmins that are released from damaged tissue post-trauma. Our data suggest that mtDAMPs may also contribute to systemic immune dysfunction post-injury by prolonging the lifespan of hyperactive neutrophils that are tolerant to secondary stimulation [59].

## Figures and Tables

**Table 1:** Patient demographics.

Characteristics	Patients (n = 73)
Age, years (range)	41 (19–95)
Gender, (M:F)	65:8
Time to pre-hospital sample, minutes post-injury (range)	40 (14–60)
ISS (range) ^#^	25 (9–66)
Admission GCS score (range)	10 (3–15)
Mechanism of injury	
Fall, n (%)	9 (58)
A/P, n (%)	18 (25)
Blunt, n (%)	4 (12)
RTC, n (%)	42 (5)
ICU-free days (range)	20 (0–30)
Hospital-free days (range)	9 (0–29)
Mortality, n (%)	8 (11)

Data are expressed as mean (range), unless otherwise stated. ^#^ Data for ISS was available for 69 patients. A/P, assault/penetrating; GCS, Glasgow Coma Scale; ICU, intensive care unit; ISS, Injury Severity Score; RTC, road traffic collision.

## Data Availability

The raw data supporting the conclusions of this article will be made available by the authors on request.

## Supplementary Materials

The following supporting information can be downloaded at: https://www.mdpi.com/article/10.3390/cells14100754/s1. Figure S1. Gating strategy to determine mitochondrial membrane potential of neutrophils by JC-1 staining. Figure S2. Gating strategy to measure activation of caspase 3 in neutrophils. Figure S3. Measurement of reactive oxygen species (ROS) production by a luminol-amplified chemiluminescence assay. Figure S4. Gating strategy for the measurement of neutrophil phagocytic activity. Figure S5. Gating strategy for the measurement of CD16 expression on the neutrophil surface. Figure S6. Gating strategy of Annexin V and propidium iodide staining (PI) to measure neutrophil apoptosis. Figure S7. Effect of traumatic injury on the protein expression of the Bcl2 family members A1 and Bax in neutrophils. Figure S8. Endotoxin contamination is not responsible for the mitochondrial-derived damage associated molecular pattern (mtDAMP)-induced extension in neutrophil life-span. Figure S9. A greater proportion of CD16^LOW^ neutrophils exhibit Annexin V positivity when compared to CD16^HIGH^ neutrophils. Figure S10. Effect of mitochondrial-derived damage associated molecular pattern (mtDAMP) treatment on Bax protein expression in neutrophils. Figure S11. Z-DEVD-FMK pre-treatment reduces CHX-induced activation of caspase 3. Figure S12. Endotoxin contamination is not responsible for the mitochondrial-derived DNA (mtDNA)-induced extension in neutrophil life-span. Figure S13. Signalling through formyl peptide receptor-1 is not implicated in the mtDAMP-induced delay of neutrophil apoptosis caused by treatment with the protein synthesis inhibitor cycloheximide. Figure S14. Pre-treatment with the formyl peptide receptor-1 inhibitor Cyclosporin H (CsH) does not induce neutrophil apoptosis nor does it rescue neutrophils from cycloheximide (CHX)-induced apoptosis.

## Abbreviations

The following abbreviations are used in this manuscript:

**Abbreviation**	**Definition**
Act D	actinomycin D
APACHE	Acute Physiology and Chronic Health Evaluation
APC	allophycocyanin
ATP	adenosine triphosphate
AUC	area under the curve
BBATS	Brain Biomarkers after Trauma Study
Bax	Bcl-2-associated X protein
BSA	bovine serum albumin
CM	complete medium
CHX	cycloheximide
CsH	cyclosporin H
FPR-1	formyl peptide receptor-1
GCS	Glasgow Coma Scale
GM-CSF	granulocyte macrophage colony stimulating factor
GPS	glutamine, penicillin, streptomycin
HBSS	Hank’s balanced salt solution
HCL	hydrochloric acid
HCs	healthy controls
HRP	horseradish peroxidase
IL	interleukin
ISS	Injury Severity Score
JC-1	5,5′,6,6′-tetrachloro-1,1′,3,3′-tetraethylbenzimidazolylcarbocyanine iodide
Mcl-1	myeloid cell leukaemia 1
MFI	mean fluorescence intensity
MtDAMPs	mitochondrial-derived damage-associated molecular patterns
MtDNA	mitochondrial-derived DNA
MODS	multiple organ dysfunction syndrome
MOF	multiple organ failure
NAD+	β-nicotinamide adenine dinucleotide
PBS	phosphate buffered saline
PMA	phorbol 12-myristate 13-acetate
ROS	reactive oxygen species
RT-PCR	real-time PCR
RT	room temperature
SDS	sodium dodecyl sulphate
TGF-β	transforming growth factor-beta
TBST	tris-buffered saline containing tween
TNF-α	tumour necrosis factor-alpha
