# Supplementary figures and images for: Major Traumatic Injury and Exposure to Mitochondrial-Derived Damage-Associated Molecular Patterns Promotes Neutrophil Survival Accompanied by Stabilisation of the Anti-Apoptotic Protein Mcl-1
Source: Cells. 2025 May 21;14(10):754. doi: 10.3390/cells14100754 (PMC12109945; doi:10.3390/cells14100754)

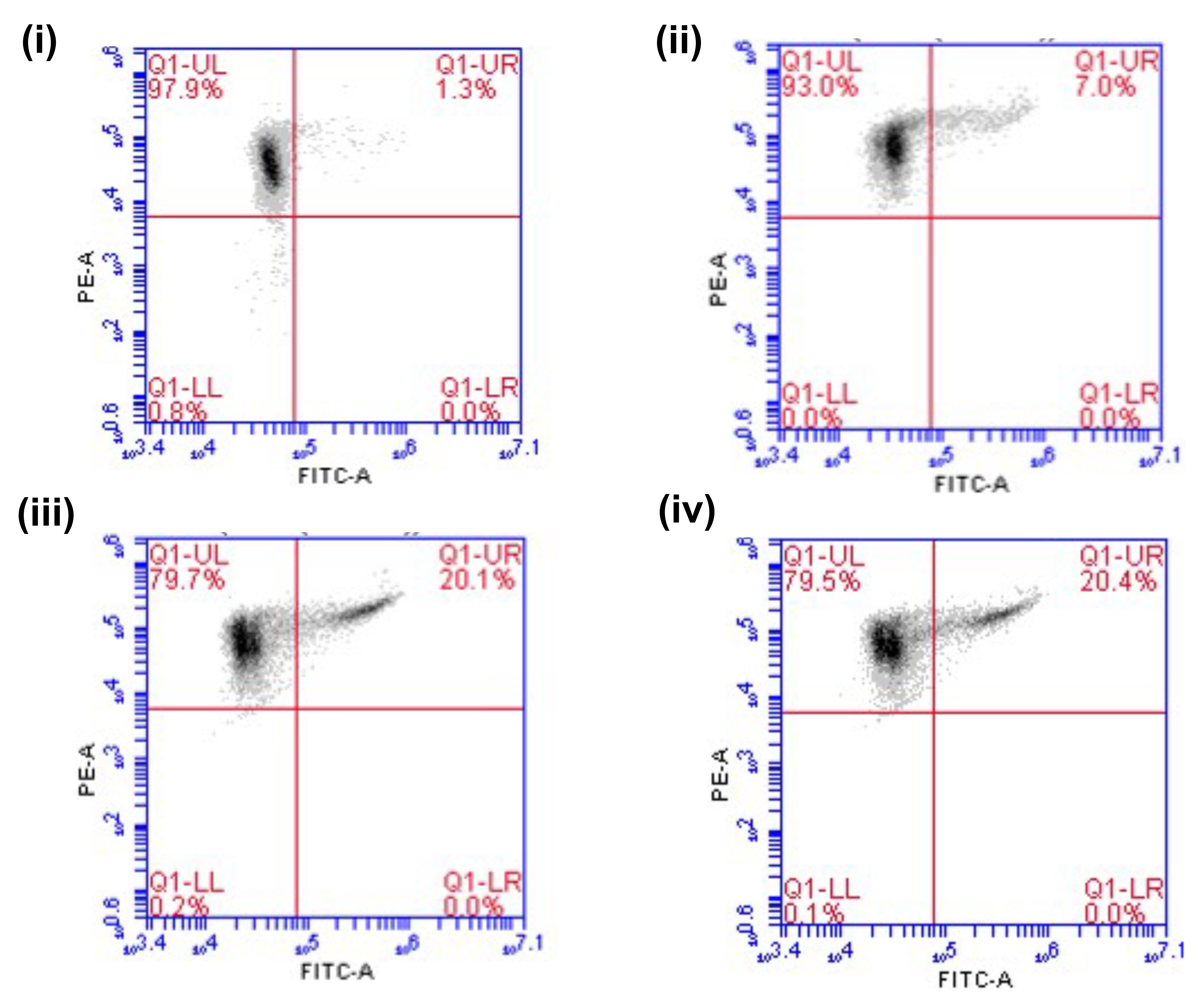

Supplement: Supplementary file 1 [file cells-14-00754-s001.zip › Supplementary Figure 1.jpg]

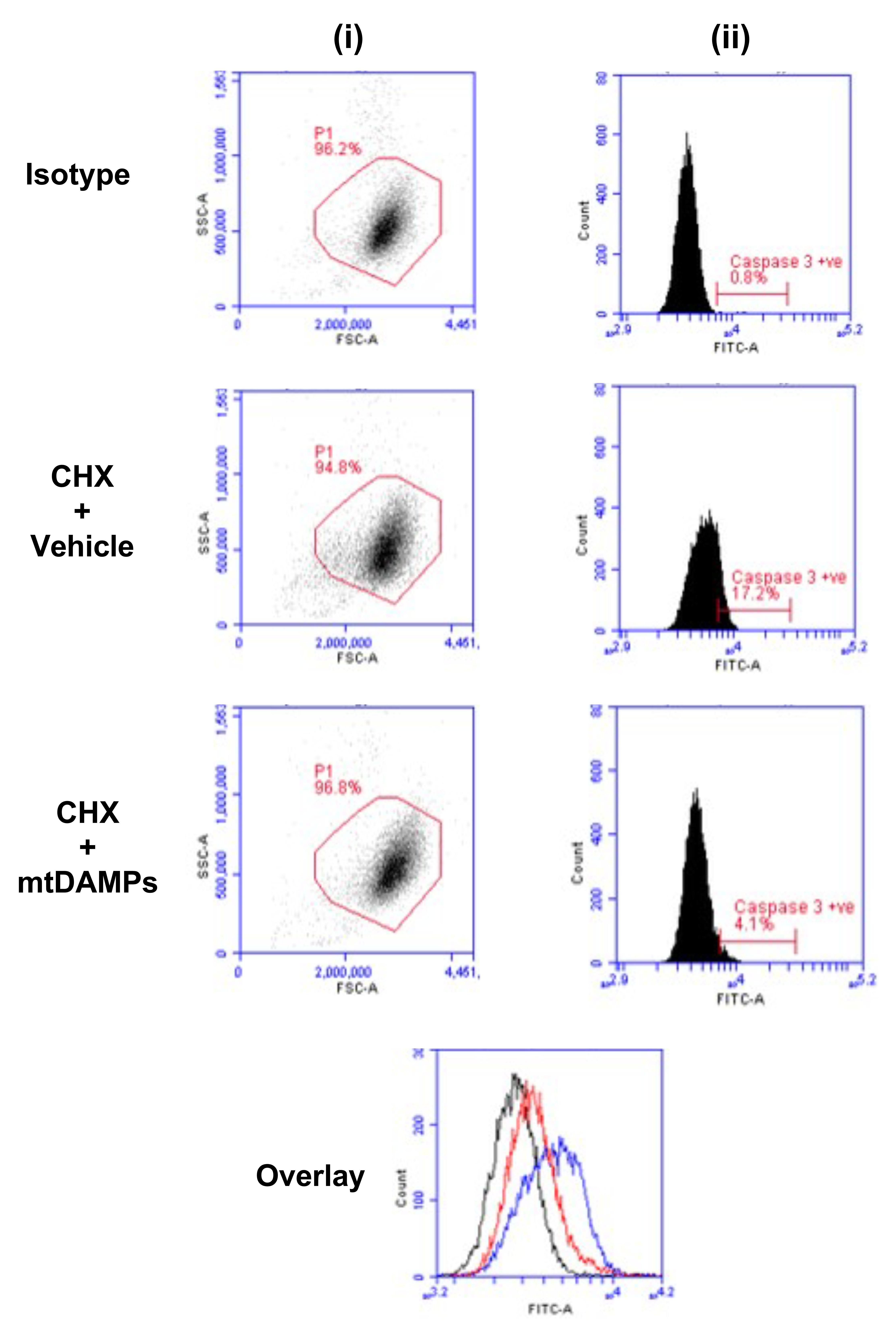

Supplement: Supplementary file 1 [file cells-14-00754-s001.zip › Supplementary Figure 2.jpg]

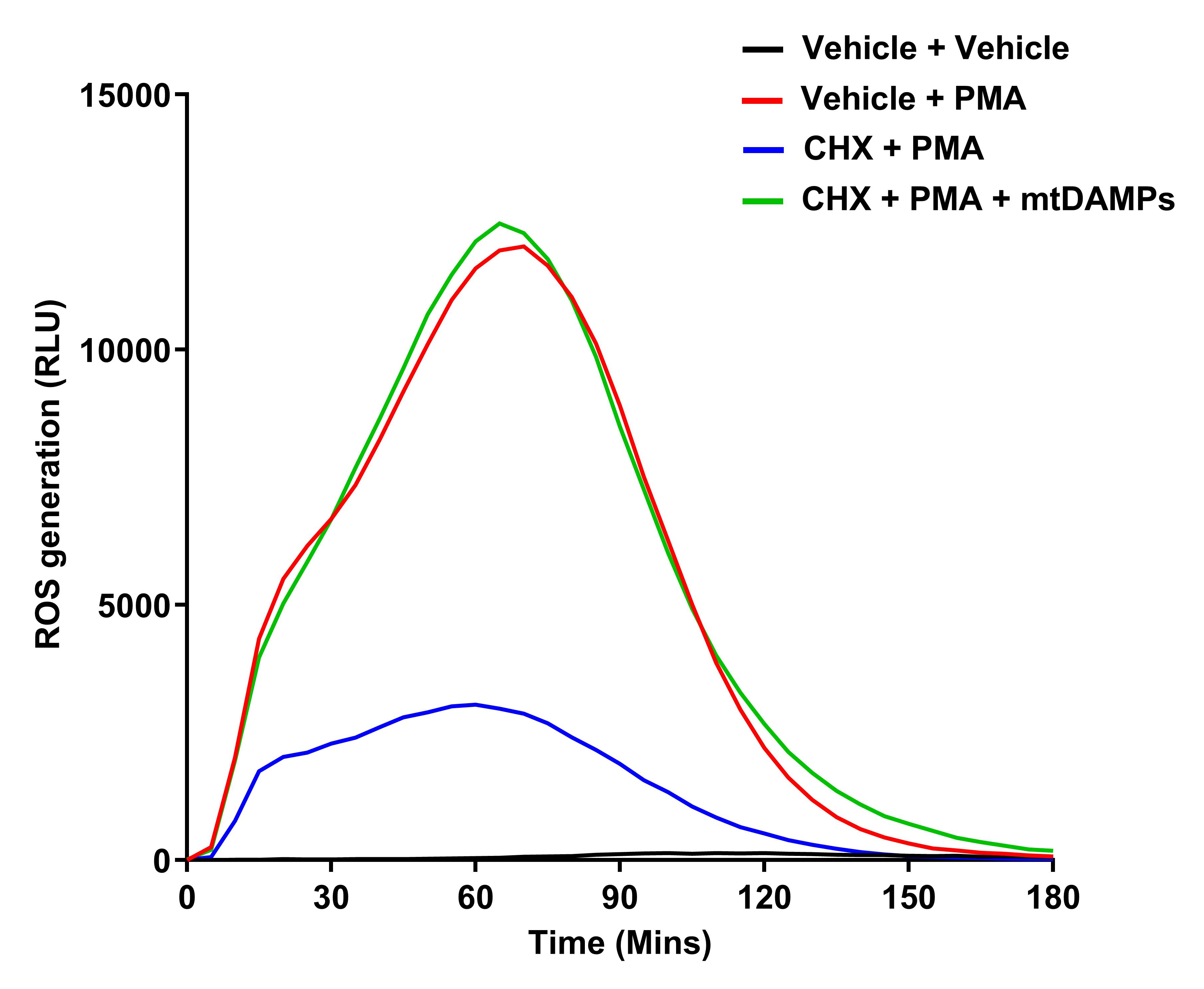

Supplement: Supplementary file 1 [file cells-14-00754-s001.zip › Supplementary Figure 3.jpg]

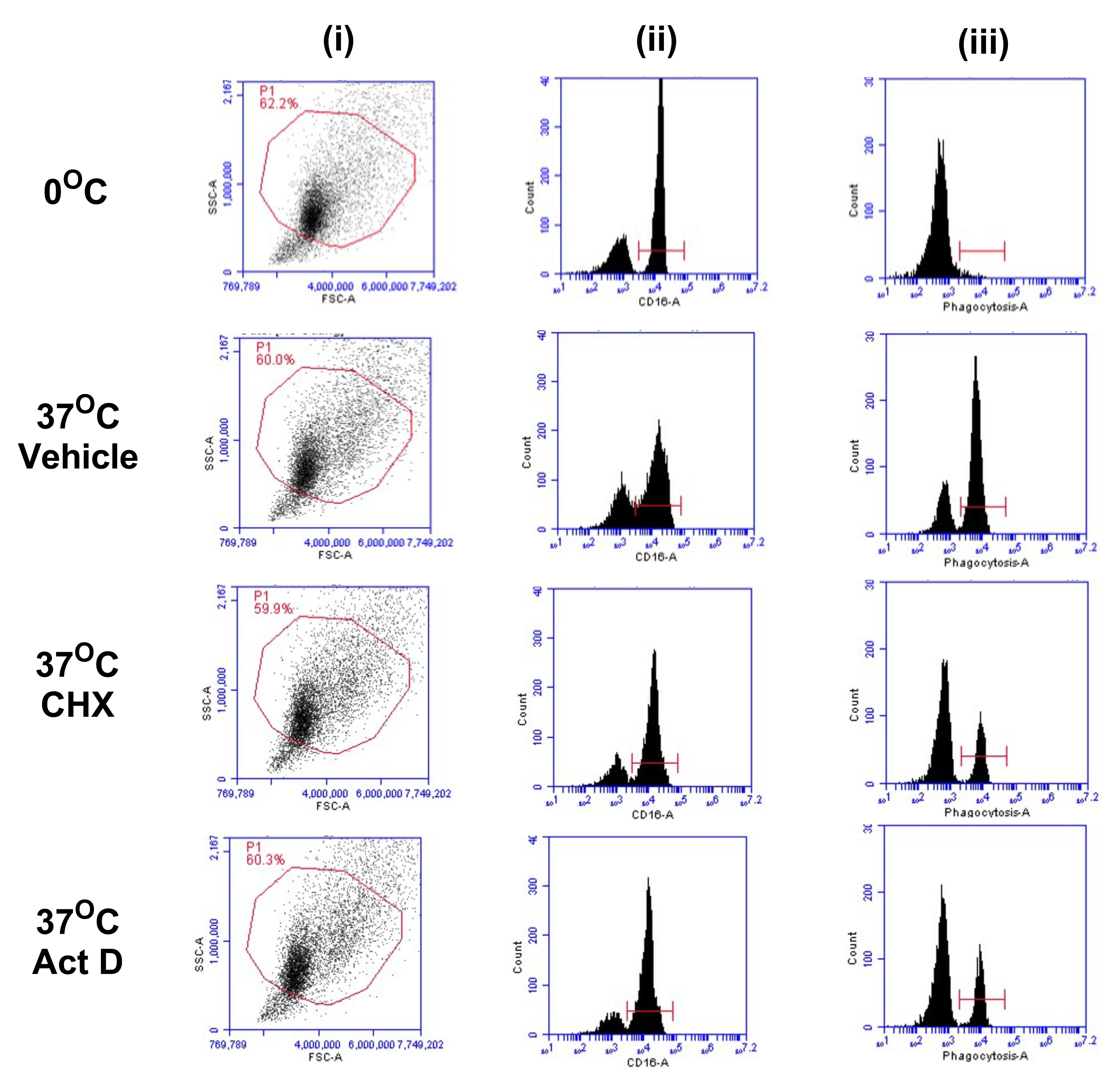

Supplement: Supplementary file 1 [file cells-14-00754-s001.zip › Supplementary Figure 4.jpg]

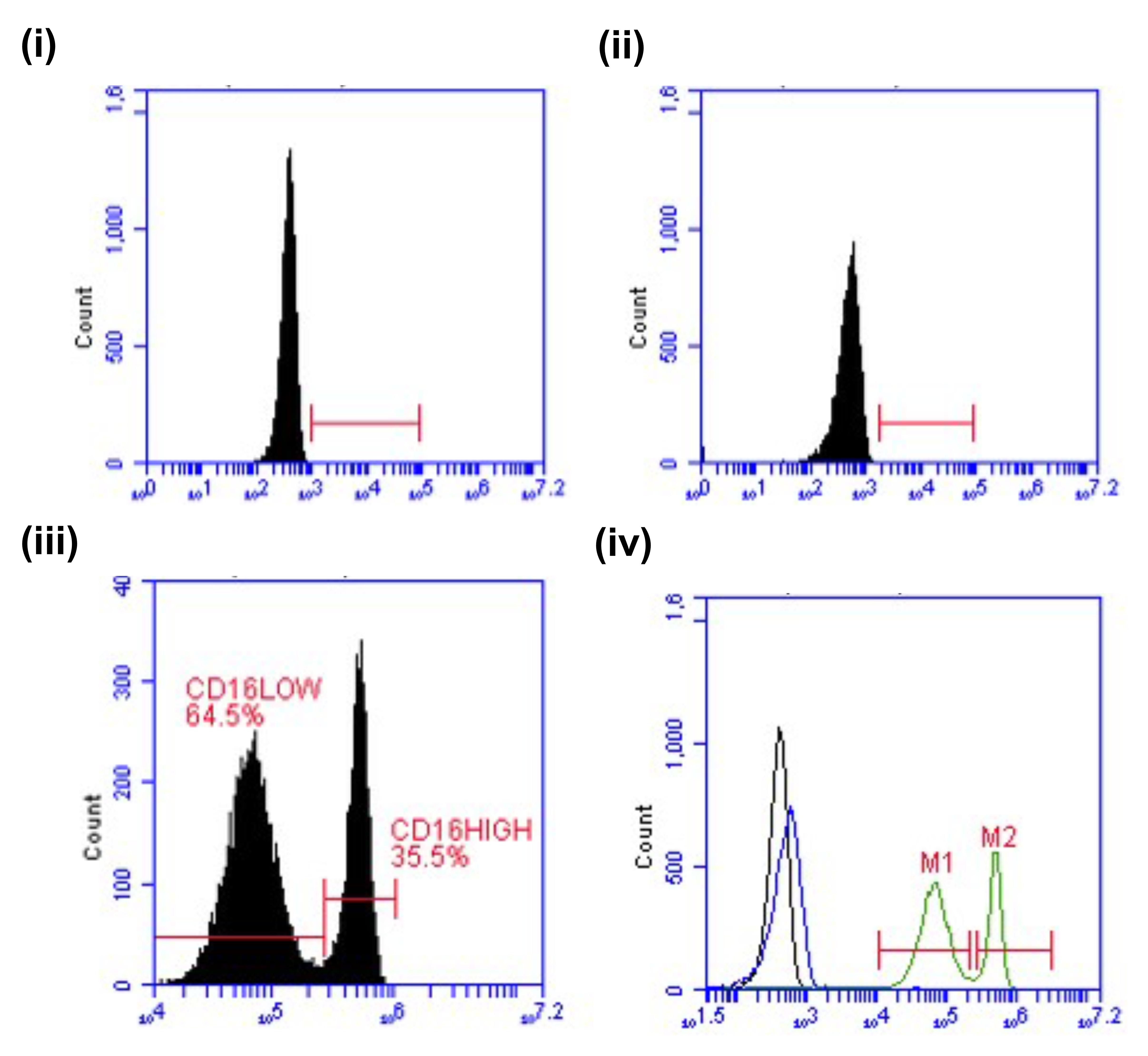

Supplement: Supplementary file 1 [file cells-14-00754-s001.zip › Supplementary Figure 5.jpg]

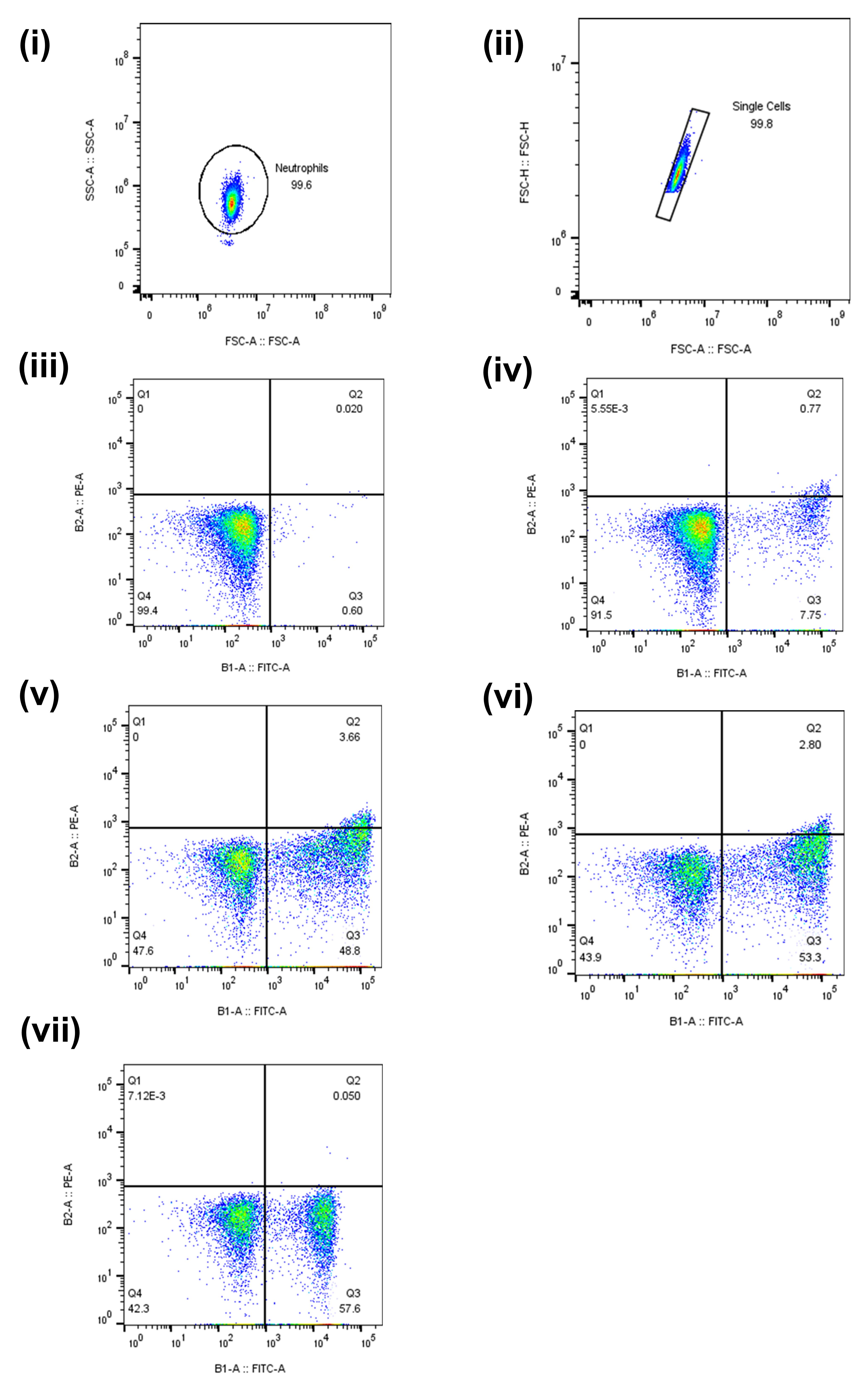

Supplement: Supplementary file 1 [file cells-14-00754-s001.zip › Supplementary Figure 6.jpg]

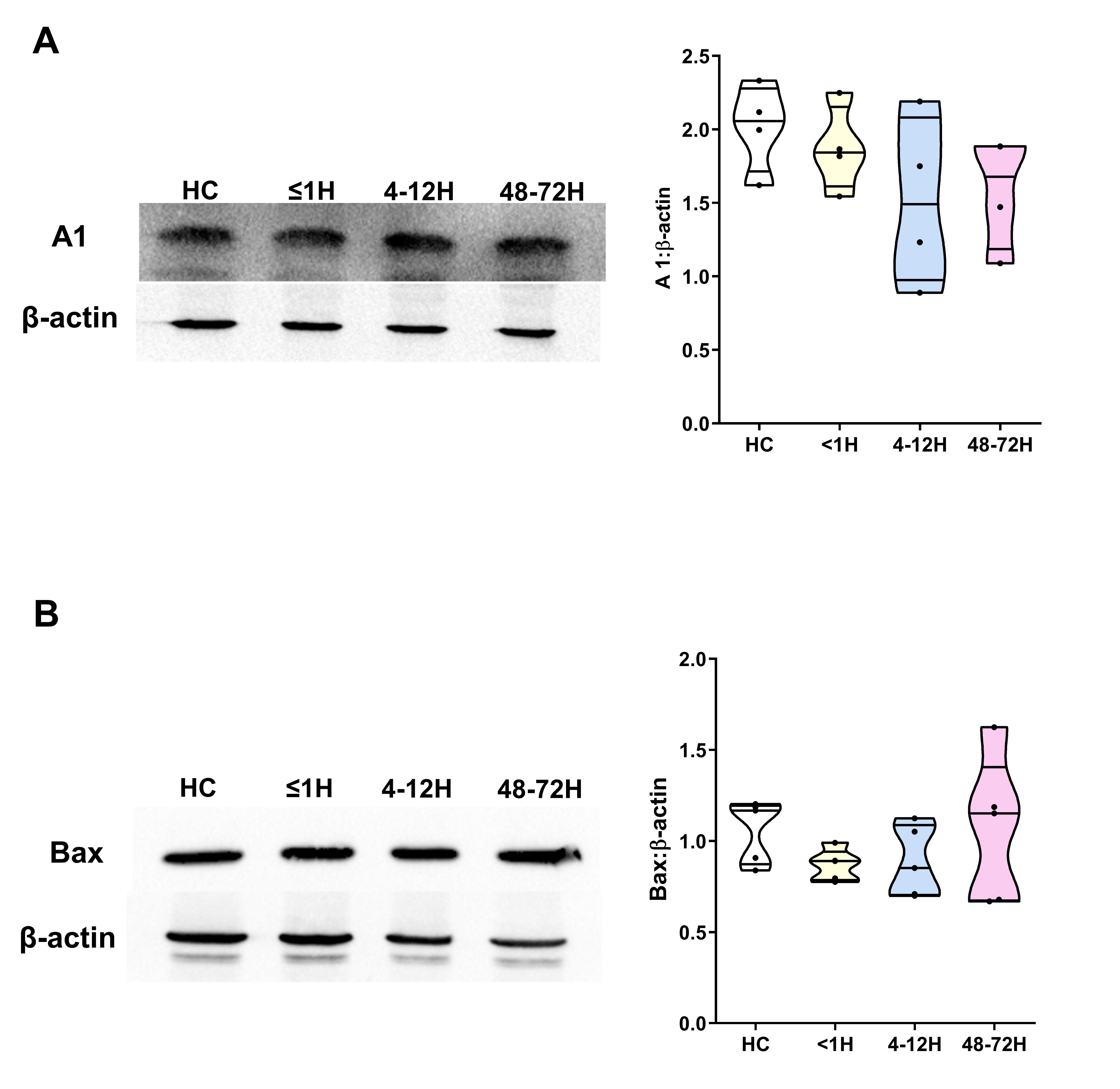

Supplement: Supplementary file 1 [file cells-14-00754-s001.zip › Supplementary Figure 7.jpg]

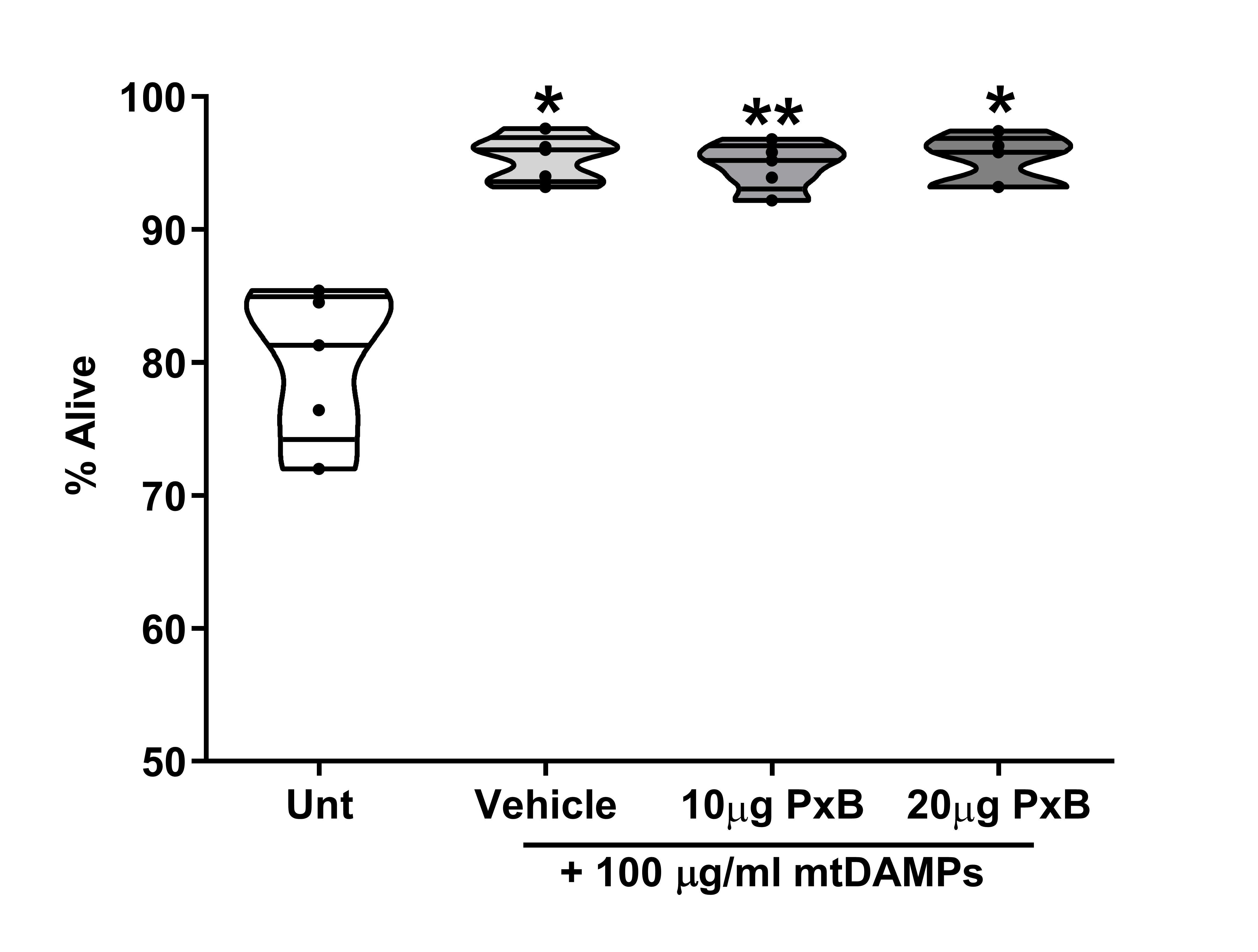

Supplement: Supplementary file 1 [file cells-14-00754-s001.zip › Supplementary Figure 8.jpg]

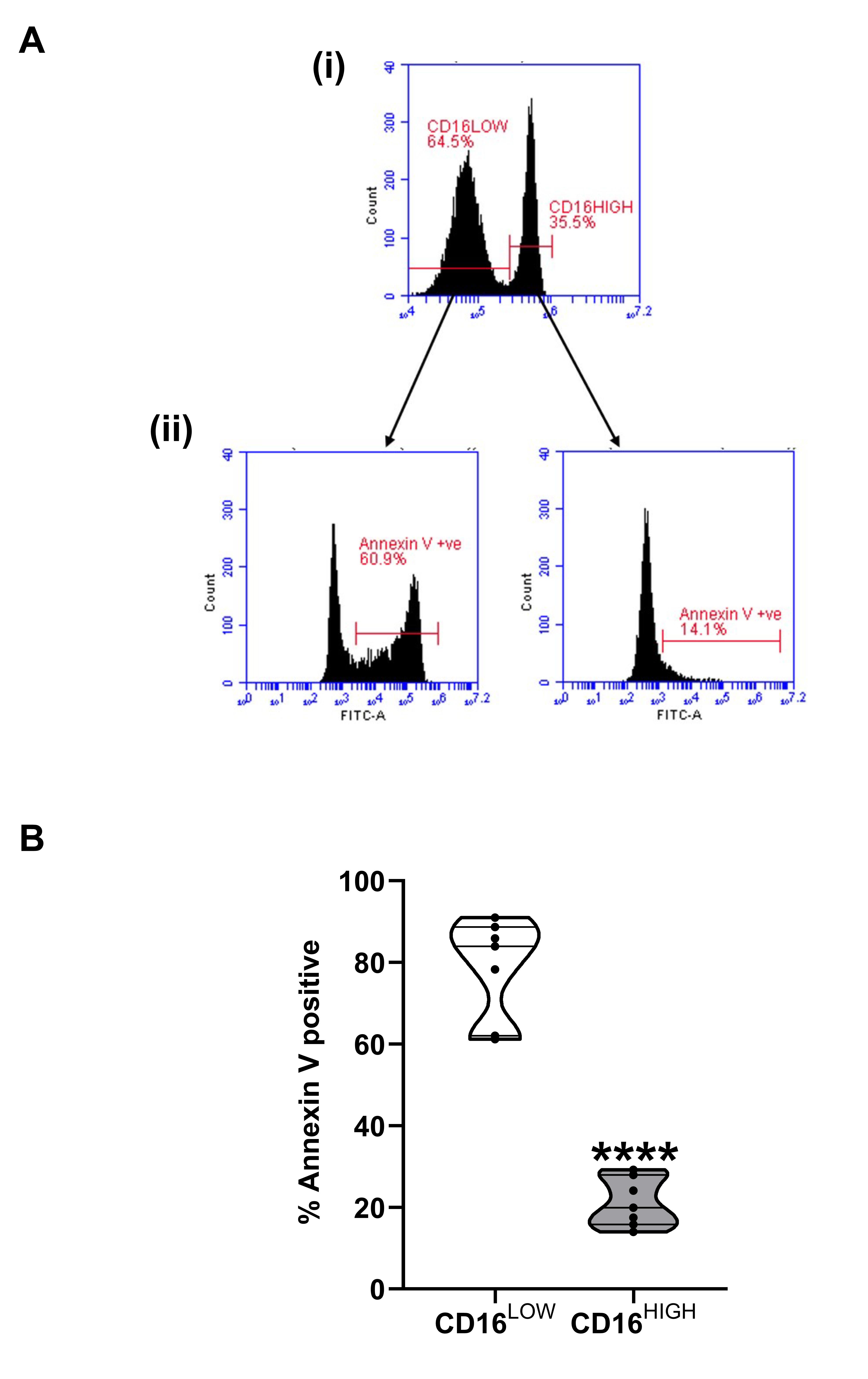

Supplement: Supplementary file 1 [file cells-14-00754-s001.zip › Supplementary Figure 9.jpg]

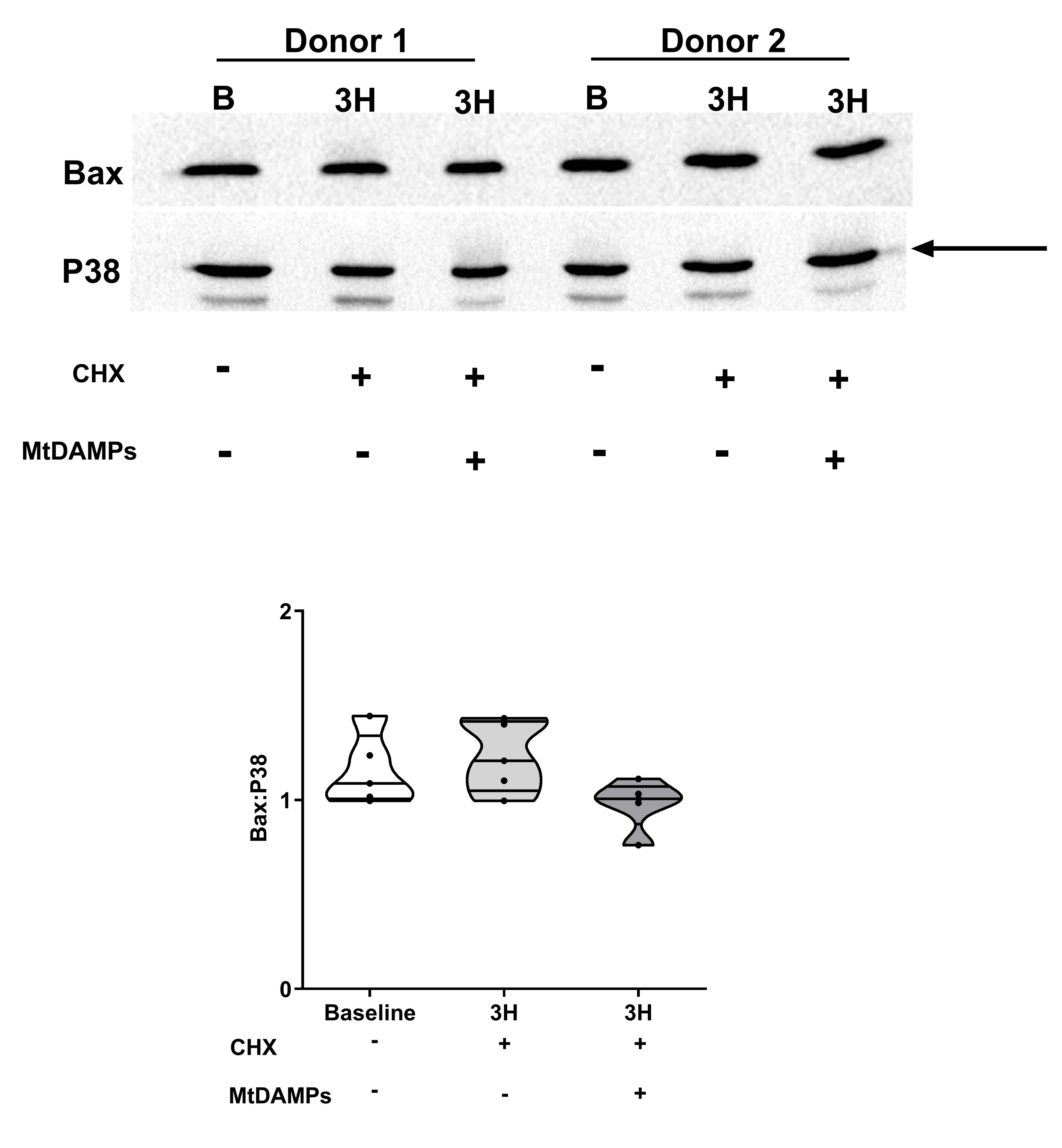

Supplement: Supplementary file 1 [file cells-14-00754-s001.zip › Supplementary Figure 10.jpg]

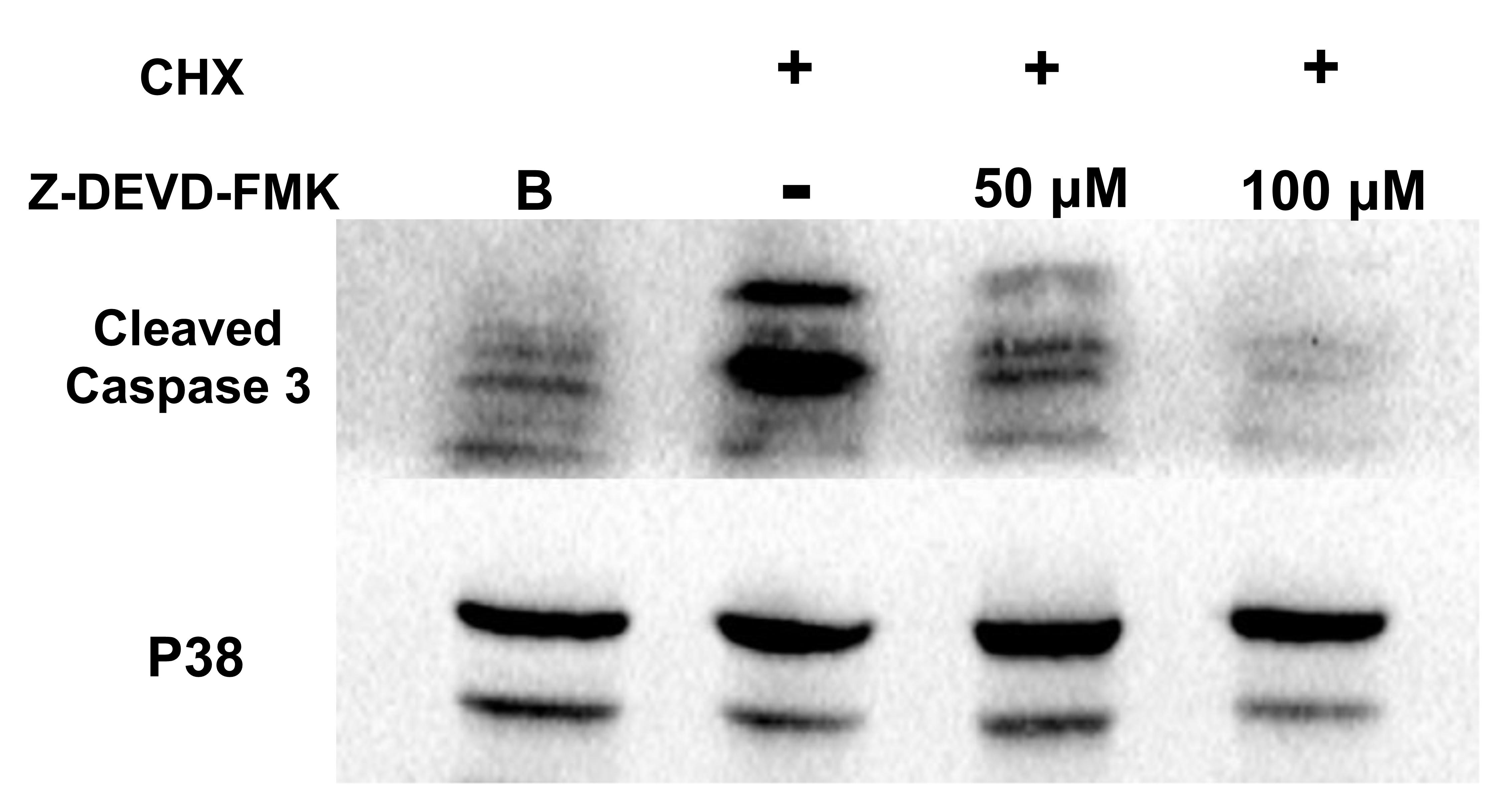

Supplement: Supplementary file 1 [file cells-14-00754-s001.zip › Supplementary Figure 11.jpg]

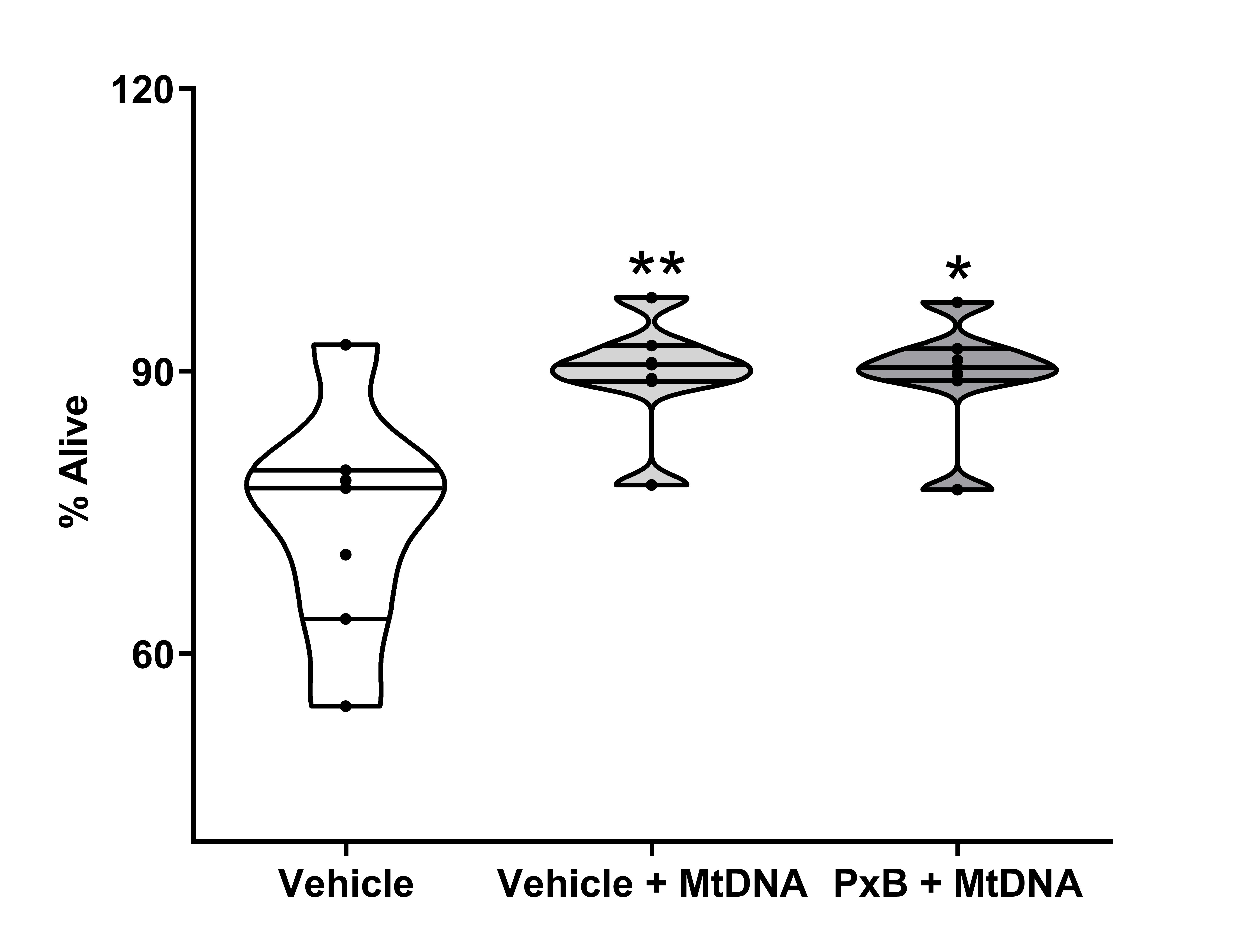

Supplement: Supplementary file 1 [file cells-14-00754-s001.zip › Supplementary Figure 12.jpg]

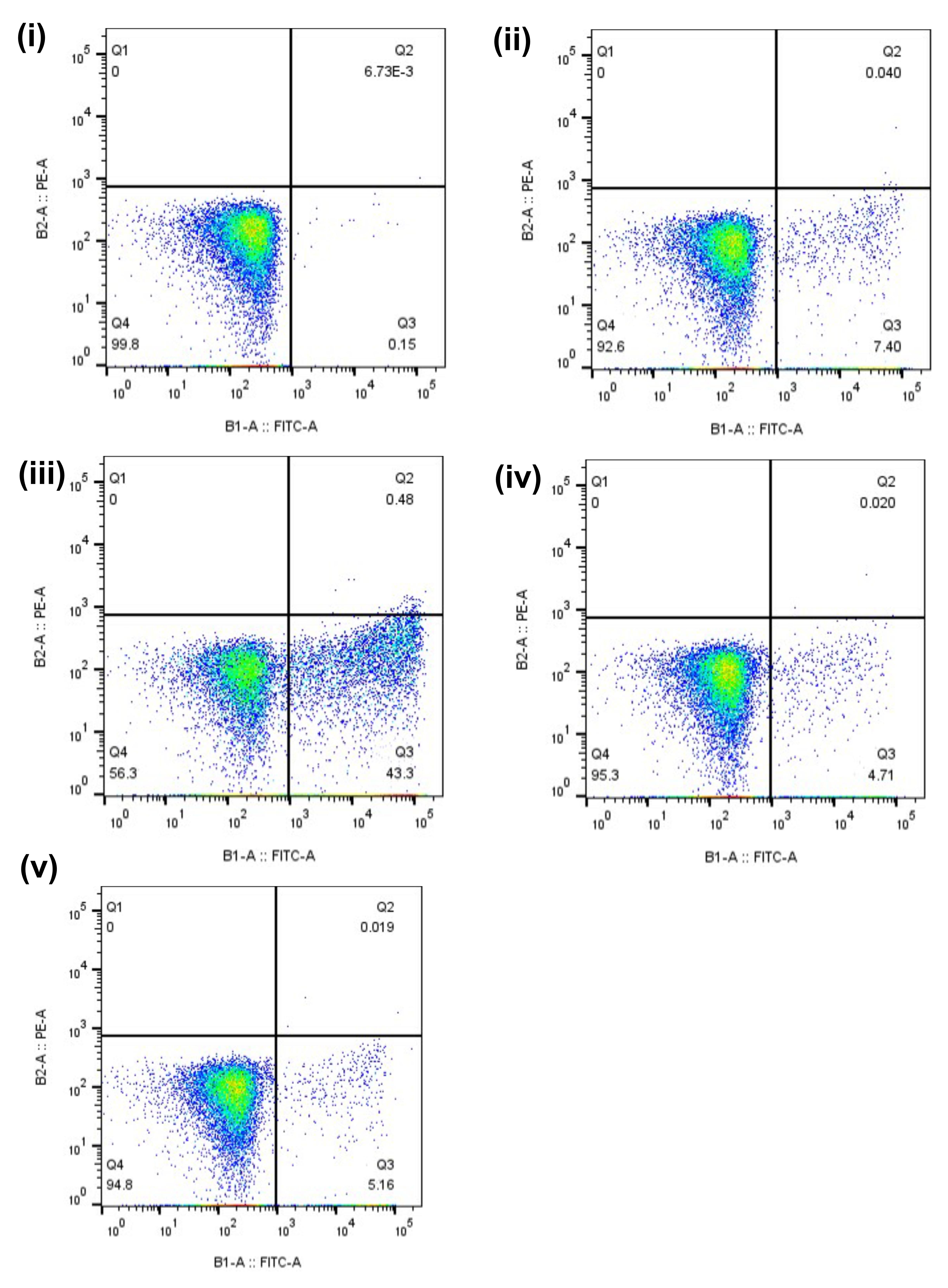

Supplement: Supplementary file 1 [file cells-14-00754-s001.zip › Supplementary Figure 13.jpg]

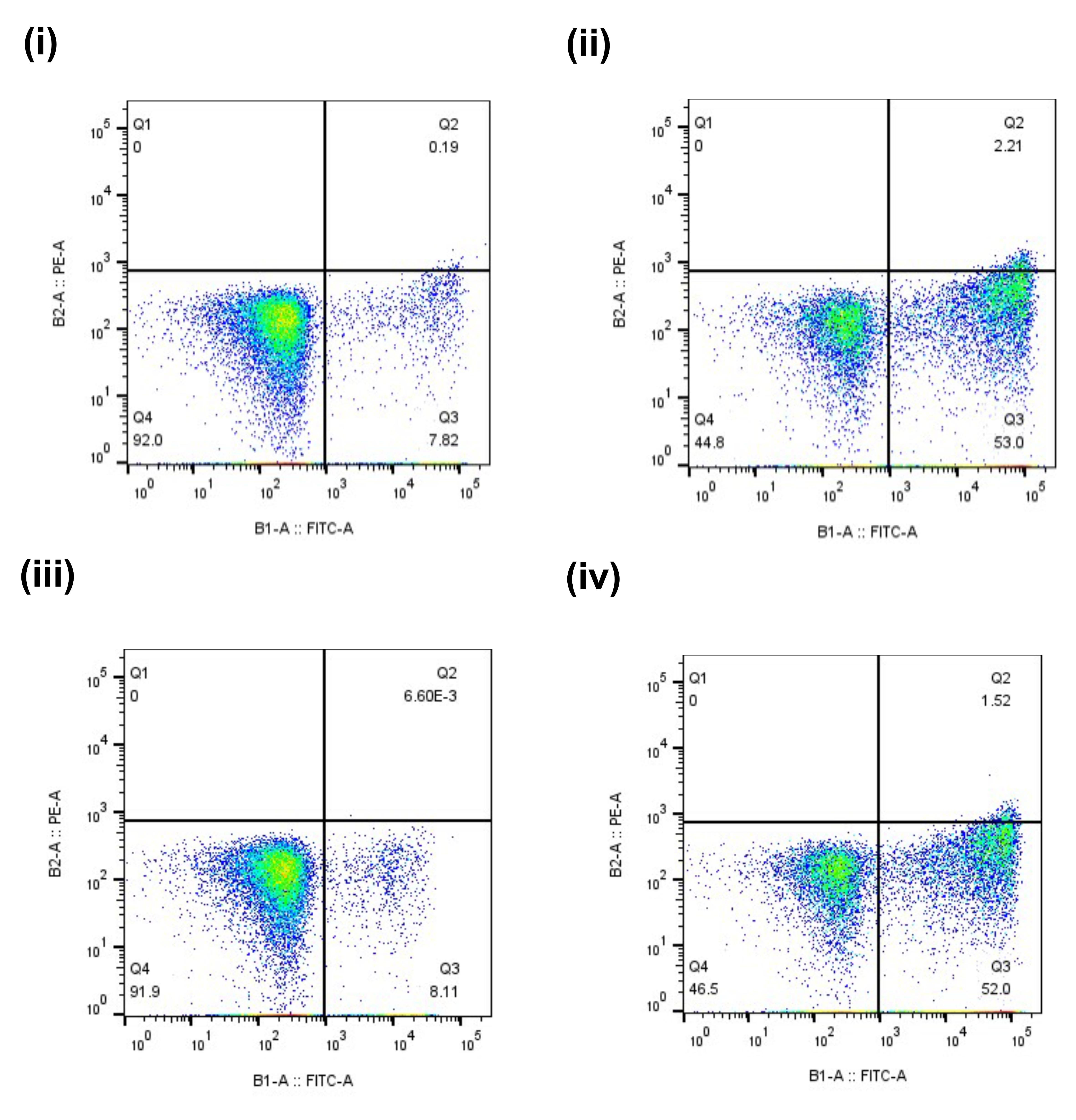

Supplement: Supplementary file 1 [file cells-14-00754-s001.zip › Supplementary Figure 14.jpg]
